# Chronic escitalopram in healthy volunteers has specific effects on reinforcement sensitivity: a double-blind, placebo-controlled semi-randomised study

**DOI:** 10.1038/s41386-022-01523-x

**Published:** 2023-01-23

**Authors:** Christelle Langley, Sophia Armand, Qiang Luo, George Savulich, Tina Segerberg, Anna Søndergaard, Elisabeth B. Pedersen, Nanna Svart, Oliver Overgaard-Hansen, Annette Johansen, Camilla Borgsted, Rudolf N. Cardinal, Trevor W. Robbins, Dea S. Stenbæk, Gitte M. Knudsen, Barbara J. Sahakian

**Affiliations:** 1 Department of Psychiatry, University of Cambridge, Cambridge, UK; 2 Behavioural and Clinical Neuroscience Institute, University of Cambridge, Cambridge, UK; 3 Neurobiology Research Unit, University of Copenhagen, Copenhagen, Denmark; 4 Department of Psychology, University of Copenhagen, Copenhagen, Denmark; 5 National Clinical Research Centre for Aging and Medicine at Huashan Hospital, MOE Key Laboratory of Computational Neuroscience and Brain-Inspired Intelligence, Institute of Science and Technology for Brain-Inspired Intelligence, Fudan University, Shanghai, China; 6 Department of Clinical Medicine, University of Copenhagen, Copenhagen, Denmark; 7 The Mental Health Services in the Capital Region of Denmark, Copenhagen, Denmark; 8 Liaison Psychiatry Service, Cambridgeshire and Peterborough NHS Foundation Trust, Cambridge, UK; 9 Department of Psychology, University of Cambridge, Cambridge, UK

**Keywords:** Human behaviour, Cognitive neuroscience

## Abstract

Several studies of the effects on cognition of selective serotonin reuptake inhibitors (SSRI), administered either acutely or sub-chronically in healthy volunteers, have found changes in learning and reinforcement outcomes. In contrast, to our knowledge, there have been no studies of chronic effects of escitalopram on cognition in healthy volunteers. This is important in view of its clinical use in major depressive disorder (MDD) and obsessive-compulsive disorder (OCD). Consequently, we aimed to investigate the chronic effect of the SSRI, escitalopram, on measures of ‘cold’ cognition (including inhibition, cognitive flexibility, memory) and ‘hot cognition’ including decision-making and particularly reinforcement learning. The study, conducted at the University of Copenhagen between May 2020 and October 2021, used a double-blind placebo-controlled design with 66 healthy volunteers, semi-randomised to receive either 20 mg of escitalopram (*n* = 32) or placebo (*n* = 34), balanced for age, sex and intelligence quotient (IQ) for at least 21 days. Questionnaires, neuropsychological tests and serum escitalopram measures were taken. We analysed group differences on the cognitive measures using linear regression models as well as innovative hierarchical Bayesian modelling of the Probabilistic Reversal Learning (PRL) task. The novel and important finding was that escitalopram reduced reinforcement sensitivity compared to placebo on both the Sequential Model-Based/Model-Free task and the PRL task. We found no other significant group differences on ‘cold’ or ‘hot’ cognition. These findings demonstrate that serotonin reuptake inhibition is involved in reinforcement learning in healthy individuals. Lower reinforcement sensitivity in response to chronic SSRI administration may reflect the ‘blunting’ effect often reported by patients with MDD treated with SSRIs. Trial Registration: NCT04239339.

## Introduction

Serotonin or 5-hydroxytryptamine (5-HT) is a monoamine neurotransmitter implicated in several cognitive and affective brain functions [1]. Drugs that target serotonin transmission, such as selective serotonin reuptake inhibitors (SSRIs) are the first-line pharmacological treatments for many neuropsychiatric disorders such as major depressive disorder (MDD), obsessive-compulsive disorder (OCD) and anxiety [2]. Understanding the modulatory role of serotonin on cognition and reinforcement learning is particularly important [3].

Many studies examining the modulatory effects of serotonin on cognition have been conducted in experimental animals [4–6]. In rats, impairing serotonin function disrupted reversal learning, whereas enhancing serotonin function improved reversal learning [4]. In marmoset monkeys, targeted neurotoxic serotonin depletion of the orbito-frontal cortex, but not of the caudate nucleus, consistently produced reversal deficits [5, 6]. Marmosets have also shown reduced reinforcement sensitivity following serotonin depletion [7].

In humans, the modulatory effects of serotonin on cognition have largely been examined through acute dietary tryptophan depletion (ATD) [8–18], or through acute SSRI administration [19–23]. ATD has been shown to affect measures of both ‘cold’ (rational and non-emotional), and ‘hot’ (social and emotional) cognition [13–18]. Specifically, ATD induces ‘waiting’ impulsivity and impulsive behaviours, impairs goal-directed behaviour and shifts behavioural control toward habitual responding in appetitive conditions, but goal-directed in aversive conditions [10–12]. Effects of ATD have also been seen on reinforcement behaviour [13], reversal learning [9, 14], learning and memory [15], affective and social cognition [9, 16, 17] and moral judgement [18]. Studies examining the acute administration of SSRIs have shown impaired probabilistic learning [19, 20], and impaired cognitive flexibility [20], but increased long-term memory recall [21], emotion recognition [22], and harm aversion for moral judgements [23]. One study showed that response inhibition improved with SSRI administration [20], whereas another showed no effect [19]. Taken together, a wide range of cognitive functions is affected by serotonin modulation in healthy volunteers.

Given that SSRIs are administered chronically in the treatment of neuropsychiatric disorders, it is particularly important to understand the long-term effects of SSRI administration on cognition. Currently, only a few studies have examined SSRIs administered sub-chronically, over approximately 7 days [24, 25]. Short-term administration of antidepressants may ameliorate the negative biases in information processing that are often present in mood and anxiety disorders [24]. A recent study examined both the acute and short-term effects of SSRIs. The results showed that acute administration did not affect reinforcement learning, but short-term administration resulted in increased learning from punishment, with reduced learning from reward [25]. However, there was no statistical difference in performance between the acute and short-term administration, and therefore these results must be interpreted with caution. In addition, studies with patients with MDD have shown that SSRIs impair learning from negative feedback, while having negligible effect on learning from positive feedback [26]. These findings demonstrate the difficulty in understanding the modulatory role of SSRI on various cognitive and motivational processes. One study gave a tryptophan-rich diet to middle-aged healthy volunteers for 19 days and showed that emotional bias to negative stimuli was reduced [27].

Understanding the acute effects of SSRIs on cognitive processes in healthy volunteers and patients with MDD is complex. This may be due to the differing possible pre- and post-synaptic actions [28]. In addition, there is some evidence that the neuroplasticity effects of SSRIs emerge only after more chronic administration (14–21 days) [29, 30]. As such, the chronic administration of SSRI may provide more robust results. Importantly, chronic SSRI administration is an experimental model that better mimics a treatment model of MDD. In addition, to our knowledge, no studies have examined the more chronic effects of SSRIs on a wide range of cognitive measures.

Escitalopram is the active S-enantiomer of the racemic SSRI citalopram (RS-citalopram) [31]. By removing the R enantiomer and only containing the pure active S enantiomer the effects of the drug are improved [28]. For example, there are no higher dose restrictions, and it also makes the lowest dose more efficacious [28]. In addition, Escitalopram shows very high selectivity for the serotonin transporter and is thus the best choice for testing pharmacologic actions of SSRIs [28, 31]. Moreover, escitalopram is an effective treatment for moderate-to-severe major depressive disorder (MDD) and is one of the best-tolerated SSRIs [28, 31].

In the present study, we used a double-blind placebo-controlled design to examine the effects of the SSRI escitalopram administered on average for 26 days, on a comprehensive set of measures of ‘cold’ and, ‘hot’ cognition, including decision-making and computational measures of reinforcement learning. We hypothesised that SSRI treatment would affect reinforcement-related behaviour, probabilistic reversal learning, and response inhibition.

## Methods

### Participants

This pre-registered study used a double-blind placebo-controlled design with 66 healthy volunteers (Table 1) of whom 32 received 20 mg daily of escitalopram and 34 received placebo for at least 21 days (escitalopram, mean(s.d.) = 26.06(2.78) days; placebo, mean(s.d.) = 26.09(3.29) days; t(63) = 0.04; *p* = 0.97; *d* < 0.01). Participants were semi-randomised (by a staff member not involved with the participants or the data analysis) into the two groups, which were matched for age, sex and intelligence quotient (IQ) (Reynolds Intellectual Assessment Scale, RAIS). Participants aged between 18 and 45 were recruited from an established database (CIMBI) at the Neurobiology Research Unit at the University of Copenhagen. The study was pre-registered on clinicaltrials.gov (NCT04239339). The study was approved by the Danish ethics committee for the capital region of Copenhagen, Denmark (H-18038352) and all participants gave written informed consent. Participants underwent a medical screening prior to enrolment in the study to ensure they were eligible for inclusion. The study was conducted between May 2020 and October 2021. Exclusion criteria are detailed in the Supplementary Material.

**Table 1:** Demographics.

	Placebo (*n* = 34)	Escitalopram (*n* = 32)	*t* or *χ*^2^	*p*	Cohen’s *d* or phi
Age	25.76 (6.64)	24.25 (5.56)	*t* = 1.00	0.32	*d* = 0.25
Sex	22 (64.71%) Females	21 (65.63%) Females	*χ*^2^ = 0.01	0.94	*φ* = 0.01
IQ	111.00 (9.60)	112.22 (9.30)	*t* = −0.52	0.60	*d* = 0.13
Reaction time	550.22 (45.65)	539.89 (59.90)	*t* = 0.79	0.43	d = 0.19

### Questionnaires

Participants completed a comprehensive set of self-report questionnaires which, among others, assessed depressive symptoms, anxiety, impulsivity, compulsivity and personality traits. Questionnaires were completed at baseline, before medication administration and a subset were repeated at the cognitive visit after at least 21 days. Finally, a subset were repeated again one week after the cognitive visit, once the medication administration had ceased. The full list of questionnaires completed at each visit is provided in Supplementary Material*.*

### Neuropsychological tests

At baseline, participants completed the CANTAB Reaction Time task (RTI) and RAIS to assess IQ. At the cognitive visit after at least 21 days, participants completed an extensive neuropsychological test battery assessing ‘cold’ and ‘hot’ cognition including reinforcement learning. The outcome measures from the tests were categorised into specific cognitive domains a priori (pre-registration; NCT04239339). The cognitive domains included learning, inhibition, executive function, reinforcement behaviour, social cognition, emotion recognition, memory, attention, and decision-making. The neuropsychological tests included the Probabilistic Reversal Learning Task, the Interleaved Stop-Signal Go/No-Go Task, the Three-Dimensional Intra-Extra Dimensional Set Shifting Task, Cambridge Neuropsychological Test Automated Battery (CANTAB) Paired Associates Learning, CANTAB Spatial Working Memory, CANTAB Rapid Visual Information Processing, EMOTICOM Moral Judgement Task, EMOTICOM Intensity Morphing Task, EMOTICOM Emotion Recognition (Eyes) Task, EMOTICOM Ultimatum Game, EMOTICOM Cambridge Gambling Task, and the Sequential Model-Based Model-Free Task (MBMF). More detailed descriptions of the tasks are provided in Supplementary Material and Table S1.

### Experimental procedure

After participants gave their written informed consent to take part, they underwent screening for somatic illness including a medical examination, blood screening for somatic disease, an ECG, and screening for the presence of psychiatric conditions using the Mini-International Neuropsychiatric Interview, Danish translation version 6.0.0 [32]. Included participants were then semi-randomised to receive an effective clinical dose of escitalopram (20 mg daily in capsules of 10 mg) or placebo in identical capsules manufactured and distributed by the Capital Region Pharmacy, for three to five weeks. The participants and the investigators involved in the data acquisition and data analysis were blinded to the intervention type until completion of data analysis.

Participants received oral and verbal instructions by blinded medical personnel for taking escitalopram including possible side effects. They were instructed to, in the morning, take 10 mg daily for three days and 20 mg daily from the fourth day until the last day of examination when the participants were scheduled for the cognitive visit and MRI scanning. The MRI analysis is presented in a separate article. Prior to the cognitive visit, the participants completed several self-report questionnaires to evaluate their psychological state.

To verify treatment compliance, the capsule container was inspected at visits and a blood sample was taken from the participants both at the halfway point and at the cognitive visit, typically in the morning (Fig. S7). In addition, participants completed a medication logbook daily, which was checked at the follow-up assessment.

Participants were instructed to take the drug capsule after the blood sample, to ensure measurement of steady-state serum escitalopram levels. At the cognitive visit, participants completed a number of neuropsychological tests and repeated a set of the questionnaires. After the cognitive visit participants were instructed to take 10 mg daily for five days and then discontinue use. One week after the cognitive visit, participants completed the final set of questionnaires. A medical professional was involved in participant oversight and regular contact was made.

At the end of the study, participants were asked whether they thought they received escitalopram or placebo. In response, 53% of participants in the escitalopram group correctly guessed that they received escitalopram, whereas 15.6% of participants in the placebo group guessed they received escitalopram. Comparison of the two groups showed a significant difference in the ability to correctly detect group membership (*χ*^2^ (1, *N* = 65) = 10.46, *p* = 0.01 [two sided]). The ability to guess the correct allocation in the escitalopram group was at chance level.

### Data analysis

All statistical analyses were conducted in R, version 4.1.1 (R Foundation for Statistical Computing).

#### Biochemical analysis

The concentration of serum escitalopram was analysed using ultra-high performance liquid chromatography/tandem-mass spectrometry (UPLC-MS/MS) (Filadelfia Epilepsy Hospital, Denmark).

#### Questionnaires

All questionnaire group comparisons were conducted using two-tailed *t* tests in R. Multiple comparisons were conducted using the Benjamini-Hochberg [33] false discovery rate (FDR) with *q* = 0.05 for each questionnaire. The *p*-values reported are uncorrected.

#### Neuropsychological tests

The group comparisons for the neuropsychological tests were conducted using linear regression models allowing for unequal variances with the *gls* function from the R package *nlme*. Age, sex, IQ and reaction time at baseline were included as covariates in the analysis. Multiple comparisons were conducted according to the pre-registered cognitive domains using the Benjamini-Hochberg false discovery rate (FDR) with *q* = 0.05. The *p*-values reported are uncorrected.

The Sequential Model-Based/Model-Free (MBMF) task was analysed according to previously published literature [34]. A generalised linear mixed-effects regression analysis of group behaviour data was performed using the *lme4* package in R. First-stage choice (stay or switch from previous trial) was modelled by independent predictors of previous reward (reward or no reward), previous transition type (rare or common), and all interactions. The terms of interest were the main effect of reward (i.e., the model-free term), a reward × transition-type interaction effect (i.e., the model-based term), and the reward x group and reward × transition-type × group interaction effects.

Finally, for the Probabilistic Reversal Learning Task, we used an innovative computational modelling approach, by fitting families of hierarchical Bayesian reinforcement learning models to trial-by-trial task data [35, 36]. Model comparison was conducted between four models using a bridge-sampling estimate of the marginal likelihood using the *bridgesampling* [37] function in R. Model 1 included a reward learning rate, punishment learning rate and reinforcement sensitivity; Model 2 included a reward learning rate, punishment learning rate, reinforcement sensitivity and stimulus stickiness, Model 3 included a combined learning rate, reinforcement sensitivity and stimulus stickiness; and Model 4 used an experience weighted approach [38] which includes learning rate, inverse temperature and experience weight. In Model 4, learning from reinforcement is modulated by an “experience weight” for a stimulus; the experience weight for a stimulus is updated every time it is chosen, and its change over time is governed by a decay factor. In this model, the softmax inverse temperature was also a parameter able to vary.

We analysed the differences in parameter values between groups by first calculating group mean differences (MDs) per parameter. The 90% highest density intervals (HDIs) of the posterior distribution per MD were then calculated and inspected to check whether they included zero (evidence for no difference between groups). Full details of model formulation, model fitting, and parameter recovery are provided in the Supplementary Material*.*

## Results

### Demographics

The analysis confirmed that the two groups were well matched and there were no significant differences in age, sex or IQ. In addition, there were no group differences for reaction time on the CANTAB RTI at baseline.

### Biochemical analysis

The biochemical analysis confirmed that participants in the escitalopram group strictly adhered to the medication self-administration schedule, as evidenced by stable escitalopram levels above 20 nmol/L. Figure S7 shows serum escitalopram levels taken at the cognitive visit.

### Questionnaires

There were no significant group differences, after correction, between the placebo and escitalopram groups on any of the baseline questionnaires. The full results are reported in Table S2.

When examining differences between the change scores on the questionnaires between baseline and cognitive visit, the escitalopram group had significantly lower scores on the Changes in Sexual Functioning (CSFQ-14) Questionnaire, corresponding to higher dysfunction on dimension 5 (Orgasm/Ejaculation (t (42.25) = 2.68, *p* = 0.01, Cohen’s *d* = 0.68) and phase 3 (Orgasm/Completion *t*(42.25) = 2.68, *p* = 0.01, *d* = 0.68)). There was no sex difference for the change score (dimension 5 Orgasm/Ejaculation (*t*(64) = −1.15, *p* = 0.25, *d* = 0.28) and phase 3 Orgasm/Completion (*t*(64) = −1.15, *p* = 0.25, *d* = 0.28)). Full results are reported in Table S3. There were no other significant group differences between the placebo and escitalopram groups on any of the questionnaires conducted only at the cognitive visit (Table S4).

There were no group differences between the placebo and escitalopram groups on any of the follow-up questionnaires. The full results are reported in Table S5.

### Neuropsychological tests

#### Reinforcement learning

##### Standard PRL results

The analysis for the standard PRL measures showed no group differences. Descriptive measures are displayed in Table 2.

**Table 2:** Standard PRL and MBMF measures.

Task	Outcome measure	Placebo (*n* = 34)	Escitalopram (*n* = 32)
Probabilistic Reversal Learning Task	Stage 1 Mean Errors	0.88 (2.13)	2.28 (3.63)
	Stage 2 Mean Errors	5.82 (6.76)	6.88 (6.01)
	Stage 1 Switch Probability	0.44 (1.26)	0.72 (1.05)
	Stage 2 Switch Probability	0.76 (1.37)	0.88 (0.91)
Sequential Model-Based/Model-Free Task	Reward Common Transition	0.91 (0.29)	0.92 (0.28)
	Reward Rare Transition	0.88 (0.32)	0.81 (0.40)
	No Reward Common Transition	0.73 (0.44)	0.74 (0.44)
	No Reward Rare Transition	0.88 (0.33)	0.87 (0.34)

##### PRL modelling

**Fig. 1:**
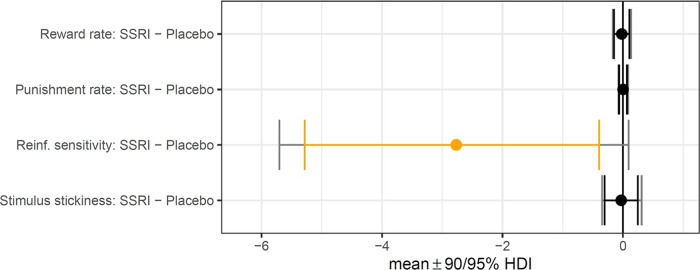
Computational modelling results from the Probabilistic Reversal Learning (PRL) Task. Error bars in orange indicate credible differences in posterior distributions between placebo and escitalopram groups for which the 90% highest density interval (HDI) excluded 0.

Hierarchical Bayesian modelling revealed that the escitalopram group had lower reinforcement sensitivity compared to the placebo group at the credible difference level of 90% (MD = −2.77 [90% HDI, −5.29 to −0.40]). There were no significant group differences for the reward learning rate (MD = −0.02 [90% HDI −0.15 to 0.11]), punishment learning rate (MD < 0.01 [90% HDI −0.07 to 0.07]) or stimulus stickiness parameters (MD = −0.03 [90% HDI −0.31 to 0.25]). The results are represented in Fig. 1 and the model comparison is presented in Table S6.

##### Model-Based/Model-Free Task

**Fig. 2:**
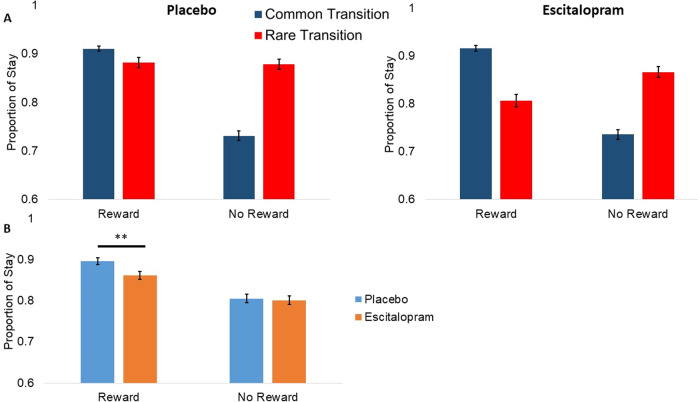
Proportion of ‘stay’ choices of the Sequential Model-Based/Model Free Task. **A** The proportion of first-stage stay choices is shown as a function of outcome of the previous trial for each group, separately for trials following common and rare transitions. The figure shows that both the placebo and escitalopram groups responded in a model-based way. **B** The proportion of first-stage stay choices is graphed as a function of outcome of the previous trial for each group averaged across transition type. The figure shows that the escitalopram group were less likely to stay following a reward compared to the placebo group. This result suggests lower reinforcement sensitivity in the escitalopram group. The error bars represent ±1 SEM.

Both the placebo and escitalopram groups performed the MBMF task in an equivalently model-based way (Fig. 2A), as demonstrated by a significant Reward × Transition-Type interaction (estimated coefficient = 0.88, *p* < 0.01, 95% CI [0.33 to 1.43]) and a non-significant Reward × Transition-Type × Group interaction (estimated coefficient = −0.17, *p* = 0.29, 95% CI [−0.48 to 0.14]). However, the escitalopram group again exhibited lower reinforcement sensitivity (Fig. 2B) compared to the placebo group (Reward × Group interaction estimated coefficient = −0.34, *p* < 0.01, 95% CI [−0.57 to −0.11]). Descriptive measures are displayed in Table 2, the full results are displayed in Table S7.

#### ‘Hot’ cognition

There were no significant group differences for any of the ‘hot’ cognitive outcomes that survived multiple comparisons. The full results are displayed in Table S8. Due to technical issues and time constraints, the exact sample size included in the analysis for each task varied slightly; the exact sample sizes are described in Supplementary Material.

#### ‘Cold’ cognition

There were no significant group differences for any of the ‘cold’ cognitive tests, therefore escitalopram had no effect on the measures of attention, memory, cognitive flexibility and response inhibition. The full results are displayed in Table S9. As above, exact sample sizes are in Supplementary Material.

## Discussion

To our knowledge, this is the first study to determine the effects of chronic escitalopram administration on a broad range of measures of ‘cold’, and ‘hot’ cognition, including reinforcement learning in healthy volunteers. In this double-blind placebo-controlled study, a relatively large group of healthy volunteers received either escitalopram or placebo for an average of 26 days. The novel and important finding was that escitalopram had the specific effect of reducing reinforcement sensitivity in two independent tests, but had no effects on other measures of ‘cold’ or ‘hot’ cognition.

### Reinforcement behaviour

The reinforcement sensitivity parameter, as modelled here, governs the degree to which a participant is driven by their reinforcement history [35]. Using this innovative approach, we found reduced reinforcement sensitivity in the escitalopram group in two different test paradigms, one on model-based vs model-free behaviour (Fig. 2) and the other in a standard PRL task. A previous study examining how reinforcement is influenced by serotonergic modulation showed that acute tryptophan depletion decreased reinforcement sensitivity, by impairing the representation of reward outcome value [13]. This was only the case for reward sensitivity, and there was no effect on punishment sensitivity [13]. In the present study, we did not find any effects on reward or punishment learning rates, whereas one study showed increased reward learning neural signals, specifically related to prediction error, following 2 week SSRI administration [39]. However, it is important to note the different methodologies used in these studies, which makes a direct comparison of the results difficult.

Importantly, our results are of considerable relevance when considering the patients’ experience of taking SSRIs chronically. Patients’ often report experiencing a ‘blunting’ effect [40–42]. This blunting effect has also been demonstrated for rewarding and punishing stimuli. Specifically, participants receiving 7 days of SSRI had lower neural processing of both rewarding and aversive stimuli [43]. In light of our own results, it is possible that the clinical effectiveness of SSRIs for MDD is due to this reduced negative affect. However, if indeed positive affect is also reduced, then this would lead to a more general blunting effect, as often reported by patients taking chronic SSRIs. This is supported by the present study, in which lower reinforcement sensitivity would suggest decreased control over behaviour by both rewarding and punishing stimuli. This may also be further supported by our findings that the escitalopram group had significantly higher dysfunction on the dimensions/phases corresponding to orgasm/completion on the CSFQ-14. It is possible that participants taking escitalopram experience greater sexual dysfunction due to experiencing less pleasure, which has been supported by previous reports [44]. However, this is speculative as there are other mechanisms that may explain this effect [45].

### ‘Hot’ cognition

Our results showed no effects on other measures of ‘hot’ cognition. Studies have previously shown that acute and sub-chronic SSRI intervention affects emotion recognition, specifically for recognition of fear and happiness [22]. In our chronic administration study, we did not examine emotion recognition in each emotion, but rather examined affective bias, which was not affected significantly by escitalopram. We also did not find any effects of escitalopram on moral judgements as previously reported following acute treatment [18, 23].

### ‘Cold’ cognition

Our results showed no significant effects on any measures of ‘cold’ cognition, thus contrasting with some of our previous data obtained following acute administration [20]. Previous studies manipulating serotonin acutely have shown alterations in both ‘cold’ and ‘hot’ cognitive measures [8–23]. However, it is important to note that our study examined SSRI administration over a longer time period, which has not previously been much studied in the context of human cognition. Contrasting with the present findings Skandali and colleagues [20], who used similar neuropsychological tests, showed that participants administered escitalopram acutely made more errors to criterion during Stage 1 and exhibited increased lose-shifting after misleading negative feedback in the PRL task. However, they did not conduct the same hierarchical Bayesian modelling employed in the present study. Such an analysis of the Skandali et al. [20] similarly shows reduced reinforcement sensitivity in the escitalopram group compared to placebo controls (unpublished findings). In addition, in the present study there was no effect of escitalopram on performance of the 3D-IED task, suggesting that this result may be specific to learning when there is greater uncertainty, as the 3D-IED is deterministic and the PRL is probabilistic in nature. We showed no effect of escitalopram on response inhibition, whereas Skandali et al. [20] showed that acute escitalopram improved stop-signal reaction time. However, in line with our results, Chamberlain et al. [19] showed no acute effect of the SSRI citalopram on response inhibition.

There are a number of points to note when interpreting the results from the present study. First, it is important to acknowledge the differences between acute and chronic SSRI administration. Previous literature has suggested that neuro-adaptive changes might represent homoeostatic mechanisms by which the brain regulates neurotransmission in response to the drug [46, 47]. This may suggest that the acute effects are not as robust as longer-term effects where this mechanism would have stabilised. In addition, acute administration of SSRIs does not seem to affect neuroplasticity, which does occur when administered chronically [29, 30]. Moreover, the synaptic mechanism of action for acute and chronic SSRI administration differs [28, 48]. A meta-analysis showed that within the first week of SSRI administration, 5-HT concentrations drop, which then increases over the following two weeks of administration, although this does vary slightly in different regions of the brain [48]. We chose the duration of 3 weeks because this duration is associated with clinical benefits in patients with MDD and with translational studies of neuroplasticity effects. However, we cannot rule out that neuroplasticity effects might be greater with a longer duration of escitalopram.

Second, the approaches for induction of changes in serotonin vary and this could result in the inconsistent findings. For example, previous studies manipulating serotonin acutely with different methods and using the same PRL task, showed inconsistent results using conventional behavioural measures [9, 19, 20]. It should be noted that currently there is no way to reliably determine interstitial serotonin concentrations non-invasively in humans, which means that interpretation of the manipulations must be inferential. Finally, it is likely that the escitalopram effects are less discernible in our cognitively high-performing (average IQ > 110) healthy volunteers than in patients with MDD. Studies on MDD have found that SSRI intervention often normalises abnormal neural processing [49–52], which in turn improves cognitive functioning and at a later time point, mood [52]. As healthy individuals are cognitively intact, it is possible that the effects may be different from those in patients with MDD. Differential effects on cognition and mood can be seen when studies are conducted with healthy volunteers or patients with MDD [8, 53]. As such the mechanism of SSRIs may be more restorative in MDD, which is unnecessary in healthy individuals. Given that SSRIs are chronically administered to patients with neuropsychiatric disorders, the present results are more clinically relevant than those of acute studies.

One possible limitation of the study was that there was a significant difference in guessing group allocation. However, the escitalopram group were at chance level for guessing group allocation (53% guessing correctly) and over 15% of the placebo group guessed they were on active substance. It is difficult to know how our results on guessing group allocation compares with other studies, as this measure is frequently not reported in the literature. The results are unlikely to have been affected by this, given the lack of cognitive changes and specificity of the effect.

Our results, importantly, showed a specific significant effect on reinforcement sensitivity, where escitalopram reduced reinforcement sensitivity, which may in part be explanatory for the blunting effect often reported by patients receiving chronic SSRI treatments. This study also highlights the need for future studies to examine chronic administration of SSRIs beyond 21 days. In addition, future studies should examine the chronic effects of SSRI administration on a similar extensive battery including ‘cold’, and ‘hot’ cognition, particularly reinforcement behaviour in patients with neuropsychiatric disorders such as MDD or OCD.

## Conclusion

In contrast with previous reports on the acute effects of SSRI administration, we did not find any significant effects on ‘cold’ cognitive measures after more chronic administration (mean 26 days). Using an innovative computational modelling approach, we did find significant effects specific to reinforcement learning; chronic escitalopram reduced reinforcement sensitivity compared to placebo. These novel findings provide strong evidence for a key role of serotonin in reinforcement learning. The results have important clinical implications as they may reflect the blunting effect often reported by patients with neuropsychiatric disorders receiving chronic SSRI treatment.

## Supplementary information

Chronic escitalopram in healthy volunteers has specific effects on reinforcement sensitivity Supplementary Material

CONSORT Flow Diagram
