# Supplementary material for: Chronic escitalopram in healthy volunteers has specific effects on reinforcement sensitivity: a double-blind, placebo-controlled semi-randomised study
Source: Neuropsychopharmacology. 2023 Jan 23;48(4):664–70. doi: 10.1038/s41386-022-01523-x (PMC9938113; doi:10.1038/s41386-022-01523-x)
Supplement: Supplementary file 1 — Chronic escitalopram in healthy volunteers has specific effects on reinforcement sensitivity Supplementary Material [file 41386_2022_1523_MOESM1_ESM.docx]

# Title: Chronic escitalopram in healthy volunteers has specific effects on reinforcement sensitivity.

**Authors:**

Christelle Langley^1,2*+^, Sophia Armand^3,4+^, Qiang Luo^5^, George Savulich^1,2^, Tina Segerberg^3^, Anna Søndergaard^3,6^, Elisabeth B. Pedersen^3,6^, Nanna Svart^3,4^, Oliver Overgaard-Hansen^3^, Annette Johansen^3,6^, Camilla Borgsted^3,6,7^, Rudolf N. Cardinal^1,8^, Trevor W. Robbins^2,9^, Dea S. Stenbaek^3,4^, Gitte M. Knudsen^3,6^, Barbara J. Sahakian^1,2^

**Affiliations:**

1. Department of Psychiatry, University of Cambridge, Cambridge, UK
2. Behavioural and Clinical Neuroscience Institute, University of Cambridge, Cambridge, UK
3. Neurobiology Research Unit, University of Copenhagen, Copenhagen, Denmark
4. Department of Psychology, University of Copenhagen, Copenhagen, Denmark
5. National Clinical Research Center for Aging and Medicine at Huashan Hospital, MOE Key Laboratory of Computational Neuroscience and Brain-Inspired Intelligence, Institute of Science and Technology for Brain-Inspired Intelligence, Fudan University, Shanghai, China
6. Department of Clinical Medicine, University of Copenhagen, Copenhagen, Denmark
7. The Mental Health Services in the Capital Region of Denmark, Copenhagen, Denmark
8. Liaison Psychiatry Service, Cambridgeshire and Peterborough NHS Foundation Trust, Cambridge, UK
9. Department of Psychology, University of Cambridge, Cambridge, UK

*Corresponding Author:

Christelle Langley, Herchel Smith Building for Brain and Mind Sciences, Forvie Site, Robinson Way, Cambridge, CB20SZ, [cl798@medschl.cam.ac.uk](mailto:cl798@medschl.cam.ac.uk)

+ Joint Contribution

# Supplementary Material:

## Supplementary Methods:

### Participants:

In total, 73 volunteers were recruited, but five participants dropped out due to self-reported side-effects (escitalopram=4, placebo=1), two participants did not complete the follow-up assessment due to sickness or pain during magnetic resonance imaging (MRI), and one participant was excluded due to severe brain atrophy observed from the MRI scan.

The full exclusion criteria were as follows:

1. Current or former primary psychiatric disorder as classified in DSM-V or WHO ICD-11
2. Current or former neurological disease or severe somatic disease
3. Head injury or concussion resulting in loss of consciousness for more than two minutes
4. Current use of psychoactive medication
5. Drug use other than tobacco and alcohol within the last 30 days
6. Alcohol or drug abuse
7. Use of cannabis more than 50 times
8. Use of illegal psychoactive drugs more than 10 times for each drug
9. Use of any drugs likely to influence the test results
10. Nicotine addiction
11. Allergy to the ingredients in the administered drug
12. Abnormal ECG such as prolonged QT syndrome
13. Dizzy when changing from supine to upright position (e.g. postural orthostatic tachycardia syndrome)
14. Mild hypotension (blood pressure below 100/70 mmHg) or hypertension (blood pressure above 140/90 mmHg)
15. Contraindications for MRI such as a pacemaker or other metal implants
16. Pregnancy or lactation
17. Current or past learning disability
18. Non-fluent in Danish
19. Pronounced visual or auditory impairments
20. Severe physical impairments affecting eyesight or motor performance.

### Questionnaires:

The full list of questionnaires conducted at baseline was as follows:

1. OS-FHAM – [1]
2. Edinburgh Handedness Inventory [2]
3. Beck Depression Inventory-II [3]
4. State-Trait Anger Expression Inventory- Trait [4]
5. BIS/BAS scale [5]
6. Barratt Impulsivity Scale-11 [6]
7. The Intolerance of Uncertainty Scale [7]
8. The Obsessive-Compulsive Inventory [8]
9. Changes in Sexual Functioning Questionnaire-14 [9]
10. Positive Life Events [10]
11. Stressful Life Events [10]
12. The Penn State Worry Questionnaire [11]
13. The Brief Self-Control Scale [12]
14. State-Trait Anxiety Inventory-Trait [13]
15. The Warwick-Edinburgh Mental Well-being Scale [14]
16. Mindful Attention and Awareness Scale [15]
17. Interpersonal Reactivity Index [16]
18. Profile of Mood State [17]
19. Pittsburgh Sleep Quality Index [18]
20. Brief Symptom Checklist-53 [19]
21. Norris 16 item Visual Analogue Scale [20]

The full list of questionnaires conducted at the cognitive visit was as follows:

1. Profile of Mood State [17]
2. Pittsburgh Sleep Quality Index [18]
3. Cohen’s Perceived Stress [21]
4. Beck Depression Inventory-II [3]
5. State-Trait Anxiety Inventory-State [13]
6. State-Trait Anger Expression Inventory-State [4]
7. Brief Symptom Checklist-53 [19]
8. Barratt Impulsivity Scale-11 [6]
9. The Obsessive-Compulsive Inventory [8]
10. Changes in Sexual Functioning Questionnaire-14[9]
11. The Intolerance of Uncertainty Scale [7]
12. The Penn State Worry Questionnaire [11]
13. The Brief Self-Control Scale [12]
14. Warwick-Edinburgh Mental Well-being Scale [14]
15. Mindful Attention Awareness Scale [15]
16. Verran-Snyder-Halpern Sleep Scale [22]
17. Norris 16 item Visual Analogue Scale [20]

The full list of questionnaires conducted at 1 week after the cognitive visit was as follows:

1. Profile of Mood State [17]
2. State-Trait Anger Expression Inventory- State [4]
3. Barratt Impulsivity Scale-11 [6]
4. Norris 16 item Visual Analogue Scale [20]

### Neuropsychological Tests:

Additional details regarding the neuropsychological tests and the outcome measures is supplied in **Table S1**.

#### Probabilistic Reversal Learning Task (PRL)

**
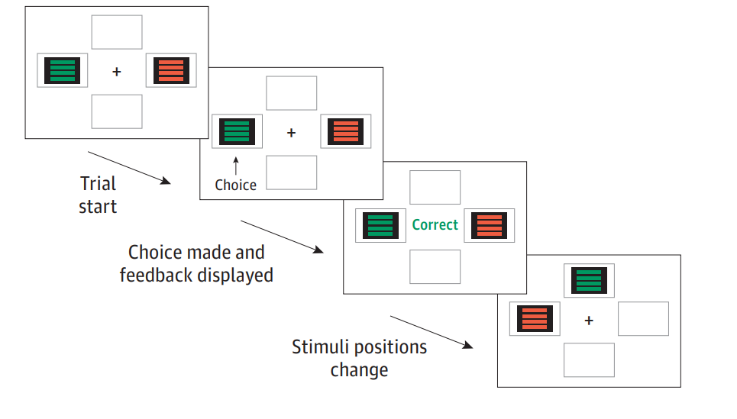
**

***Figure S1.*** *Probabilistic reversal learning task structure. Adapted from Marzuki et al., 2021 [24].*

The paradigm used here is identical to the task originally used by Murphy et al. [23]. Participants were shown two stimuli, composed of four red and four green lines (***Figure S1***), on a laptop screen. They were instructed to choose either stimulus on every trial by touching it with their finger on the screen. The task consisted of 80 trials in total and was split into acquisition and reversal phases, each consisting of 40 trials. The Acquisition phase required participants to discriminate between the optimal and non-optimal stimuli. The optimal stimulus was programmed to provide positive feedback (‘Correct’) on 80% of trials and negative feedback (‘Incorrect’) on 20% of trials. The non-optimal stimulus was programmed to provide negative feedback on 80% of trials and positive feedback on 20% of trials. The stimulus chosen by participants on the first trial was assigned as the optimal stimulus for the rest of the acquisition phase. Subsequently during the reversal phase, the positive to negative feedback ratio associated with each stimulus was reversed. In other words, the stimulus that was previously optimal became non-optimal and vice versa.

On correct trials, the word ‘Correct’ would be displayed in green, whereas on incorrect trials, the word ‘Incorrect’ would be displayed in red alongside a dissonant tone. There was no time limit for responding in each trial. The task duration was approximately 7 minutes in total.

#### Sequential Model-Based/Model-Free Task (MBMF)

**
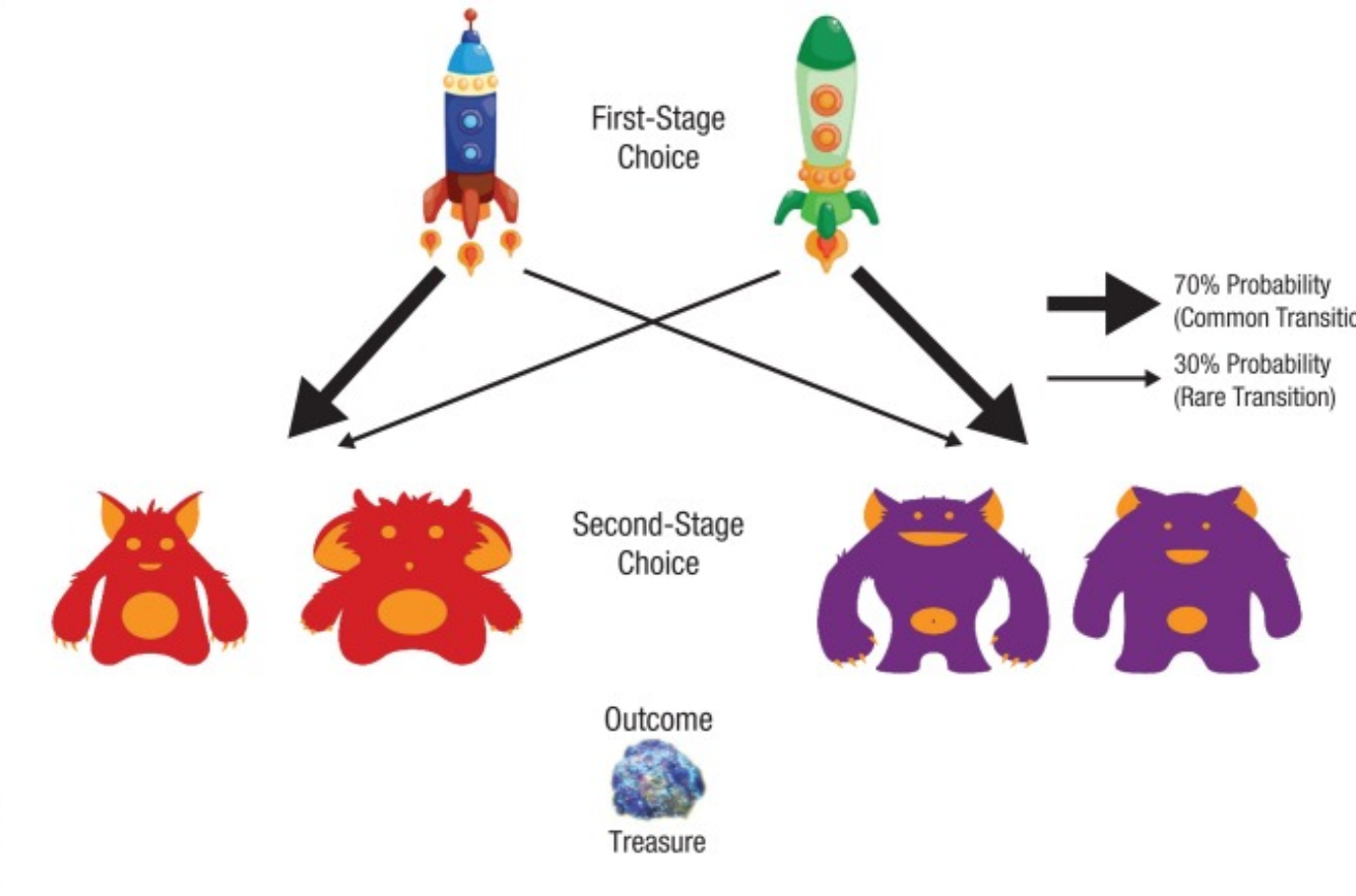
**

***Figure S2.*** *Sequential Model-Based/Model-Free task structure. Adapted from Decker et al., 2016 [25].*

The paradigm used here is identical to the task originally used by Decker et al. [25]. Participants were required to collect “space treasure” (***Figure S2***). First, they chose between two spaceship stimuli (first-stage choice). Each spaceship travelled more frequently to one planet than to the other (70% versus 30%). For example, the blue spaceship had a 70% probability of leading to the red planet (the common transition) and a 30% probability of leading to the purple planet (the rare transition). The green spaceship had the opposite probabilities (i.e., 70% chance of the purple planet and 30% chance of the red planet). On each planet, participants chose between two alien creature stimuli (second-stage choice). They were then rewarded with a picture of space treasure or with nothing (an empty circle) according to a slowly drifting probability (between 0.2 and 0.8) for each alien, with the other alien having the converse probability of yielding treasure. These shifting reward probabilities encouraged participants to explore different choices throughout the task to maximize rewards. Participants had 3 s to make each choice, followed by a 1 s animation, 1 s of reward feedback, and a 1 s intertrial interval. The full game consisted of 200 trials in four blocks separated by breaks.

Before the task, all participants completed a tutorial that conveyed the task cover story and introduced key concepts, such as probabilistic rewards and transitions, via a series of interactive example trials. The tutorial was automated to ensure that all participants received equivalent information.

#### Interleaved Stop Signal Go/No-Go Task (SS/GnG
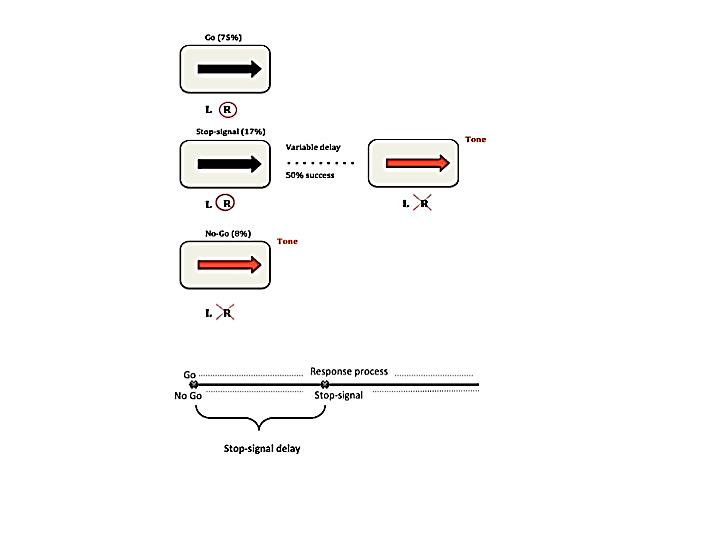


***Figure S3.*** *The interleaved Stop Signal Go/No-Go trials task structure. Adapted from Skandali et al., 2016[26].*

The paradigm used here is identical to the task used by Skandali et al. (2016) [26] and developed by Ye et al. (2014) [27]. Participants were presented with three types of trials; 360 Go trials (75%) requiring a right or left button press depending on the direction of a black arrow on the screen, 80 Stop-signal trials (17%, with ~50% successful, as the task titrates the onset of the stop signal towards 50% success) requiring cancellation of a cued button press when the black arrow turns red and 40 No-Go trials (8%) requiring the subject to withhold from pressing any button as the arrow appears red (***Figure S3***).

#### Three Dimensional Intra-Extra Dimension Set Shifting Task (3D-IED)


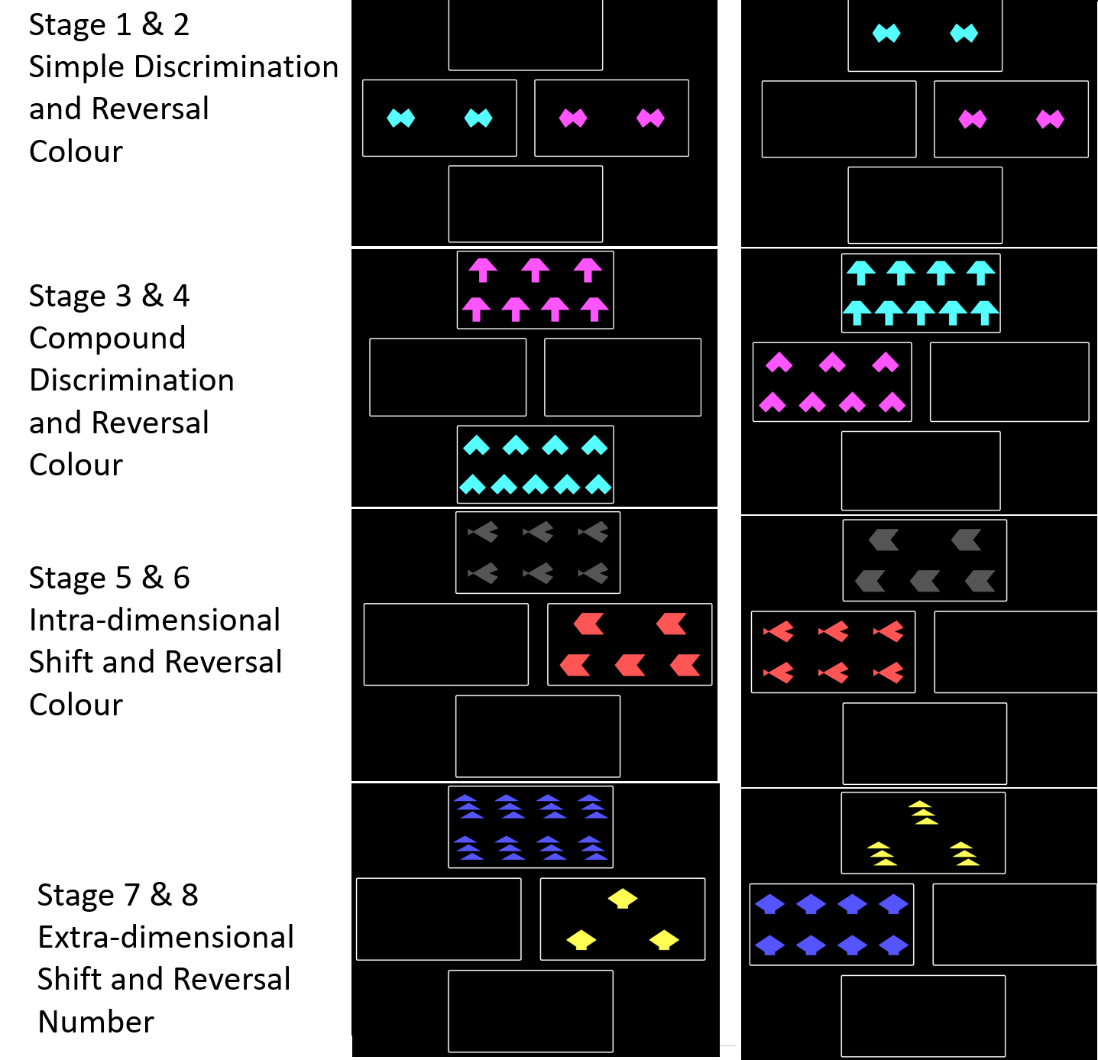


***Figure S4.*** *Three Dimensional Intra-Extra Dimension Set Shifting Task structure*.

This task was adapted from the CANTAB Intra-Extra Dimension (IED) Set Shifting Task [28, 29] The CANTAB IED is a test measuring cognitive flexibility. The task [30] initially features rule acquisition and reversal learning and then attentional set formation and set shifting. For the 3-D IED there are three dimensions of interest (shape, colour, and number; ***Figure S4***). Importantly, the learning criterion remain the same, after six correct responses, the stimuli and/or rule changes. Early in the test the shifts are intra-dimensional (ID) (colour is relevant) to establish an attentional set. This stage assesses generalisation of learning. Then there is a crucial extra-dimensional (ED) shift (number becomes relevant; attentional set-shifting), which assesses cognitive flexibility. This latter stage is followed by a final reversal of the rule. Importantly, the task is not time-limited and participants have 50 trials to reach the learning criterion.


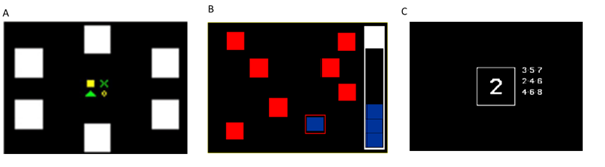


***Figure S5.*** *CANTAB Tasks:* ***(A)*** *Paired Associates Learning.* ***(B)*** *Spatial Working Memory.* ***(C)*** *Rapid Visual Information Processing.*

#### CANTAB Paired Associates Learning (PAL)

The CANTAB PAL [28, 31, 32] (***Figure S5A***) is an eight-minute test assessing visuospatial memory and learning. Boxes are displayed on the screen in a spatial array and opened by the program in a random order to display the contents. One or more boxes contain a visual pattern. The patterns are subsequently displayed one by one in the middle of the screen and the participant must select the box in which the pattern was previously presented. If the participant makes an error, the boxes are opened in the same order again. This is to remind the participant of the locations of the patterns before they attempt to remember again.

#### CANTAB Spatial Working Memory (SWM)

The CANTAB SWM [28, 33] (***Figure S5B***) is a nine-minute test assessing spatial working memory. Test performance requires the retention and manipulation of visuospatial information. Coloured boxes are shown on screen and participants must select a box with a token. The token is stored on the edge of the screen and will not appear in the same location for the rest of the trial. Therefore, returning to the same location on the next search is an error. The colour and position of the boxes used are changed from trial to trial to discourage the use of stereotyped search strategies.

#### CANTAB Rapid Visual Information Processing (RVP)

The RVP [28, 34] (***Figure S5C***) is a 10-minute test which measures sustained attention by presenting a rapid stream of digits and requiring participants to detect target sequences. A white box is displayed in the centre of the screen in which digits 2-9 are rapidly presented at 100 digits per minute. Participants are required to detect target sequences (e.g. 2-4-7, 3-5-7 or 4-6-8) and respond to this target sequence as quickly as possible.


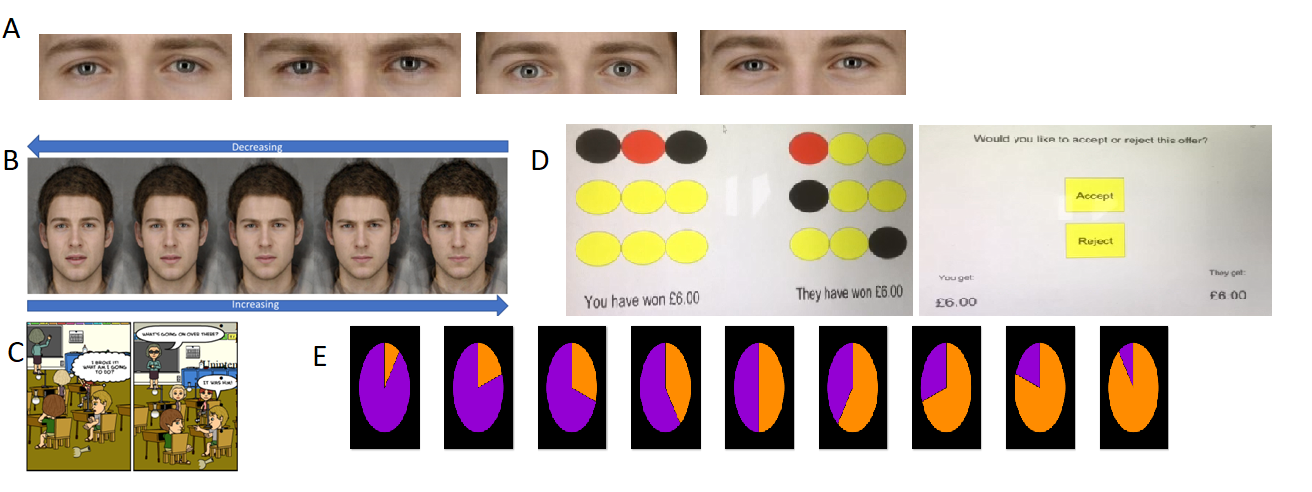


***Figure S6.*** *EMOTICOM Tasks:* ***(A)*** *Emotion Recognition Eyes.* ***(B)*** *Intensity Morphing.* ***(C)*** *Moral Judgement Task.* ***(D)*** *Ultimatum Game.* ***(E)*** *Cambridge Gambling Task.*

#### EMOTICOM Emotion Recognition Eyes

This task was designed as part of the EMOTICOM Battery [35]. In this 12-minute task, the participant is shown a series of eyes that appear on the screen briefly (***Figure S6A***), and is asked to identify the emotion (happiness, sadness, anger or fear). In the control condition, participants are asked to identify the age of a face (child, young adult, middle aged, elderly).

#### EMOTICOM Intensity Morphing

This task was designed as part of the EMOTICOM Battery [35]. The intensity morphing test (***Figure S6B***) measures the emotional intensity at which participants recognise a facial emotion. In a 10-minute test, participants are presented with faces that either increase or decrease in emotional intensity. Participants are required to respond when they either first see the emotion (increasing) or can no longer see the emotion (decreasing). There are five emotions in total—happiness, sadness, anger, fear, and disgust—and participants were explicitly told which emotion was presented.

#### EMOTICOM Moral Judgement Task

This task was designed as part of the EMOTICOM Battery [35]. In the 20-minute moral judgement task (***Figure S6C***) participants view cartoon figures depicting moral scenarios. Participants are asked to rate their levels of guilt, shame, annoyance and feeling “bad” following each of the cartoons. Half of the cartoons are portrayed as deliberate harm and half as unintended harm. Moreover, the participants were asked to rate their emotions from the perspective of both the victim and the perpetrator.

#### EMOTICOM Ultimatum Game

This task was designed as part of the EMOTICOM Battery [35]. This 12-minute task (***Figure 6SD***) assesses sensitivity to fairness and tendency to inflict punishment. Similarly to the Prisoner’s Dilemma, participants initially complete a task in which they can win money. Here they can select three balls from a choice of nine and, depending on what colours are revealed behind the balls, participants can win money. Each trial is manipulated so that the participant wins more money, the opponent wins more money, or they both win equal amounts. This money is then combined with the opponent’s total. Next, participants are informed whether they get to decide how the money is split or whether it is up to the opponent. If the opponent divides the money, the participant gets the choice to either accept or reject their offer. These offers have seven levels ranging from fair (50:50) to increasingly unfair (10:90). If the participant accepts, they each get the allotted amount, and if they reject, they both get nothing. When the participant divides, they can choose from four divisions differing in fairness (50:50, 40:60, 30:70, 20:80, and 10:90).

#### EMOTICOM Cambridge Gambling Task

This task was designed as part of the EMOTICOM Battery [35]. This 10-minute task (***Figure S6E***) was developed to assess decision-making and risk-taking behaviour, with reward and loss trials administered separately. On each trial, the participant is presented with a roulette wheel; a proportion of which is coloured purple and a proportion of which is orange. There are five different proportions, ranging from very certain to very uncertain. Participants must place a bet on the outcome they expect. A spinning pointer is then displayed, which lands on one of the colours, providing feedback for the participant. There are two conditions, loss condition and win condition which allows the separation of reward and punishment.

### Hierarchical Bayesian Modelling:

To investigate processes underlying learning and decision-making on the Probabilistic Reversal Learning Task (PRL), a family of simple reinforcement learning (RL) models were fitted to data. Modelling first involves formulating a mathematical function equipped with different parameters of interest to analyse trial-by-trial data. Model code was adapted from prior studies [24, 36].

Model 1 included three parameters: a reward learning rate parameter (*α*_rew_), a punishment learning rate parameter (*α*_pun_) and a reinforcement sensitivity parameter (*τ_reinf_*). A value (*Q*) was assigned to each task stimulus, representing the expected rewards associated with them. A high *Q* denoted a higher chance of reward associated with a stimulus, while a lower *Q* indicated a lower chance of a reward. *Q_k_* (the value for stimulus *k*) was updated on a trial-by-trial basis via prediction errors that represent the difference between the expected outcome on that trial *t*, *Q_k,t_*, and the actual outcome, *R_t_*. For example, if the expected outcome for a stimulus is 0, and selecting the stimulus on a given trial results in a reward, *Q* for that specific stimulus would increase. Larger prediction errors lead to faster updating of *Q*. On every trial (t), the value of a learning rate parameter (*α*_rew_ or *α*_pun_) determined the extent to which *Q* was adjusted according to the prediction error. Concretely, this was done according to the Rescorla–Wagner rule:

*Q_k_*_,_*_t_*_+1_ = *Q_k_*_,_*_t_* + *α*_rew_(*R_t_* – *Q_k_*_,_*_t_*), if *R_t_* = 1;

*Q_k_*_,_*_t_*_+1_ = *Q_k_*_,_*_t_* + *α*_pun_(*R_t_* – *Q_k_*_,_*_t_*), if R*_t_* = 0.

where *k* represents a specific stimulus (stimulus 1 or 2) and *t* represents the current trial. *R* was 1 following a rewarded outcome, and 0 following an unrewarded outcome. The term *R_t_* – *Q_k_*_,_*_t_* is the prediction error.

*α* values varied between 0 and 1. Values of *α*_rew_ govern sensitivity to (learning from) positive prediction errors (i.e., rewarding trials) while *α*_pun_ governs learning from negative prediction errors (punishing outcomes); the use of two learning rates allows the investigation of valence-specific learning.

Finally, *τ_reinf_* (reinforcement sensitivity) is an inverse temperature parameter, used within a softmax function to determine the probability *p* of choosing a stimulus *k* on trial *t*:

$$p_{k,t}=\frac{exp\left( \tau_{reinf}Q_{k,t} \right)}{\sum_{i=1}^{n} exp\left( \tau_{reinf}Q_{i,t} \right)}$$

*τ_reinf_* determined the extent to which participants’ actions were driven by *Q* values associated with the chosen stimulus. A high *τ_reinf_* leads to more “exploitative” behaviour, whereby a participant chooses mostly to maximise their rewards (i.e., participants strongly prefer the choice with the higher *Q* value). A low *τ_reinf_* enables more exploratory behaviour (lesser preference for the choice associated with the higher *Q* value).

*Model 2*

Model 2 was identical to Model 1 but with the addition of *τ*_stim_ (stimulus stickiness), which is an inverse temperature parameter that reflects the tendency for a participant to respond to the same stimulus chosen in a previous trial regardless of feedback received. Greater values of *τ*_stim_ denote increased tendency to ‘stick’ with a choice, while low values represent a tendency to switch away from the choice. Thus, *τ*_stim_ enabled us to account for perseverative behaviour. This parameter was added to the softmax function as follows:

$$p_{k,t}=\frac{exp\left( \tau_{reinf}Q_{k,t}+\tau_{stim}S_{k,t} \right)}{\sum_{i=1}^{n} exp\left( \tau_{reinf}Q_{i,t}+\tau_{stim}S_{i,t} \right)}$$

*S* represents whether the stimulus being considered on the current trial (*S_i_*_,_*_t_*) was the same as the one chosen on the previous trial (*S* = 1 for such a repeated choice, 0 otherwise). Thus, this model contained four parameters in total: *α*_rew_, *α*_pun_, *τ_reinf_*, and *τ*_stim_.

*Model 3*

Model 3 was as Model 1 but with only three parameters (*α*, *τ_reinf_*, *τ*_stim_), using a single learning rate for reinforcement (whether rewarded or unrewarded).

*Model 4*

Model 4 was distinct from the models described thus far. It was an experience-weighted attraction (EWA) model previously used by den Ouden et al. [37]. It contains three free parameters: *φ* (phi), *ρ* (rho), and *β* (beta). The model served to decouple acquisition (pre-reversal) and reversal via the experience decay factor parameter *ρ* that enables the balance between previous experience and new information to tip increasingly in favour of past experiences. The ‘experience weight’ (*n_c_*_,_*_t_*) of a current choice, *c*, reflects how often a stimulus has been chosen. It is updated according to *ρ*:

*n_c_*_,_*_t_* ← *n_c_*_,_*_t_*_–1_ *ρ* + 1

The intuition behind *ρ* is that over time, experience accumulated during acquisition could make reversal more difficult, leading to perseveration. *ρ* was allowed to range between 0 and 1. When *ρ* = 0, predictions are always driven by most recent outcomes, whereas when *ρ* = 1 all trials are weighted equally, leading to perseveration of responses after reversal. The value function of a choice on every trial, *v_c_*_,_*_t_* (similar to *Q_t_*), is updated according to the outcome (rewarded or unrewarded), *λ*, and the pay-off decay factor *φ*, which is equivalent to the learning rate in Model 1.

*v_c_*_,_*_t_* ← (*v_c_*_,_*_t_*_–1_ *φ* *n_c_*_,_*_t_*_–1_ + *λ_t_*_–1_) / *n_c_*_,_*_t_*

When *ρ* = 0, *n_c_*_,_*_t_* on every trial becomes 1 and therefore reduces to a standard Rescorla–Wagner model. Equivalent to models described earlier, the probability *P* of choices *c* were determined via a softmax function:

$$P\left( c_{t+1}=k \right)=\frac{e^{{\beta V}_{c=k,t+1}}}{\sum_{i=1}^{n} e^{{\beta V}_{c=i,t+1}}}$$

where, as before, the inverse temperature parameter *β* controls the extent to which choices are made according to the value function *V*. Low values of *β* lead to more exploratory choices, while high values lead to choices that serve to maximise rewards.

**Model Fitting and Parameter Estimation**

Models were fit to trial-by-trial data using a hierarchical Bayesian approach, estimating the posterior distribution of the model parameters at the individual subject and group levels. This enabled estimating parameter distributions per group while controlling for inter-subject variability in behaviour. At the top of the hierarchy, separate distributions were defined for placebo and escitalopram groups. Parameter estimations for each group were sampled from the following prior distributions:

*α*_group_, *α*_group,rew_, *α*_group,pun_, *φ*_group_, *ρ*_group_ ~ Beta(1.2, 1.2)

*τ*_group_, *β*_group_ ~ Gamma(4.82, 0.88)

*τ*_group,stim_ ~ Normal(0, 1)

Inter-subject variability, σ, was sampled from half-normal prior distributions, constrained to be positive:

*σ_α_*, *σ_α_*_rew_, *σ_α_*_pun_, *σ_τ_*_stim_, *σ_φ_*, *σ_ρ_* ~ half-Normal(0, 0.05)

*σ_τreinf_*, *σ_β_* ~ half-Normal(0, 1)

Subject-level parameters were sampled from normal distributions whose means were the group level parameter values and whose standard deviations were the inter-subject variability parameter values (restricted to the valid range for the parameter). For example, in the case of *α*_rew_:

*α*_rew,subject_ = *α*_rew,group(subject) +_ *σ_α_*_rew(subject)_

All priors were obtained from earlier studies which used identical models [24, 36]. All models were fitted to data using Markov chain Monte Carlo (MCMC) sampling implemented in RStan v2.21.2. Eight randomly initialised MCMC chains were used. Convergence of chains was confirmed using the potential scale reduction statistic R̂. A cut-off R̂ value of 1.2 [24, 36] was used to check that the chains were well mixed for each parameter.

#### Parameter Recovery

We conducted parameter recovery to verify the validity of the winning model (see Model Comparisons section below). The winning model was first used to simulate synthetic data from 100 ‘participants’. The free parameters were replaced with the mean fitted parameter values per group estimated from fitting the model to actual human data. We then ascertained whether the true parameter values could be recovered by fitting the winning model to the simulated data and checking whether the true (synthetic) parameter values fell within their recovered corresponding 95% highest posterior density intervals (HDIs).

#### Model Comparisons

Models for both tasks were compared using bridge sampling via the “bridgesampling” R package [38]. This method enables selection of the best-fitting model by accounting for the prior probability and marginal likelihood of each model (the likelihood of the data given a specific model). The marginal likelihood is calculated via the product of the likelihood of the data given a fitted model and the probability of parameters given the model, which penalises over-complex models and guards against overfitting.

#### Group Differences

Posterior distributions of parameters from the winning model was interpreted using the 95% and 90% highest posterior density intervals (HDIs), also known as the Bayesian credible interval. Parameter comparisons between groups were calculated by examining the difference between the relevant placebo group’s parameter and the corresponding escitalopram group’s parameters (escitalopram – placebo), i.e. the group mean differences per parameter. The 95% and 90% HDIs of the posterior distribution for the group mean differences were calculated and inspected to check whether they included zero (indicating no difference between groups). Multiple comparisons corrections were not applied since they are not necessary for these Bayesian techniques [39, 40].

## Supplementary Results:

### Biochemical Analysis:


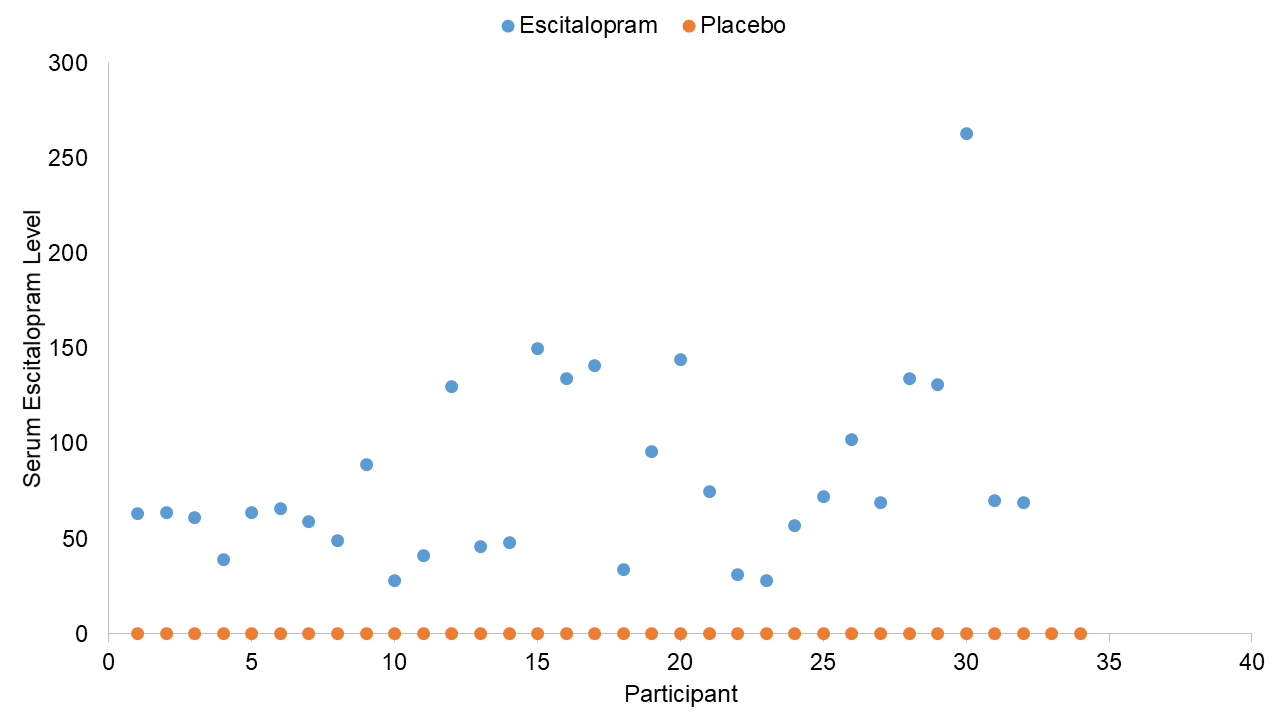


***Figure S7.*** *Serum Escitalopram Levels in nmol/L. The placebo group are represented in orange and values are 0 for the whole group. The escitalopram group are represented in blue and values are above 20 nmol/L, which indicates* increased stable escitalopram levels.

### Questionnaires:

The full analyses for the questionnaire data are displayed in ***Table S2-5.***

### Neuropsychological Tests:

The exact sample size for each task was as follows: Probabilistic Reversal Learning Task (placebo 34, escitalopram 32); Sequential Model-Based/Model Free Task (placebo 34, escitalopram 32); Interleaved Stop Signal Go/No-Go (placebo 28, escitalopram 26); 3-D IED (placebo 33, escitalopram 32); CANTAB SWM (placebo 34, escitalopram 32); CANTAB PAL (placebo 34, escitalopram 31); CANTAB RVP (placebo 34, escitalopram 32); EMOTICOM Intensity Morphing (placebo 34, escitalopram 32); EMOTICOM Emotion Recognition (placebo 34, escitalopram 32); EMOTICOM Moral Judgement Task (placebo 34, escitalopram 32); EMOTICOM Ultimatum Game (placebo 34, escitalopram 32); EMOTICOM Cambridge Gambling Task (placebo 31, escitalopram 30).

The full analyses for the neuropsychological tests are displayed in ***Table S6-9.***

## Supplementary References

1. Dam VH, Thystrup CK, Jensen PS, Bland AR, Mortensen EL, Elliott R, Sahakian BJ, et al. Psychometric properties and validation of the EMOTICOM test battery in a healthy Danish population. Frontiers in psychology. 2019;10:2660.
2. Oldfield RC. The assessment and analysis of handedness: the Edinburgh inventory. Neuropsychologia. 1971;9(1):97-113.
3. Beck AT, Steer RA, Brown G. Beck depression inventory–II. Psychological assessment. 1996.
4. Spielberger CD. State‐Trait anger expression inventory. The Corsini encyclopedia of psychology. 2010:1.
5. Carver CS, White TL. Behavioral inhibition, behavioral activation, and affective responses to impending reward and punishment: the BIS/BAS scales. Journal of personality and social psychology. 1994;67(2):319.
6. Patton JH, Stanford MS, Barratt ES. Factor structure of the Barratt Impulsiveness scale. Journal of Clinical Psychology. 1995;51:768–764.
7. Buhr K, Dugas MJ. The intolerance of uncertainty scale: Psychometric properties of the English version. Behaviour research and therapy. 2002;40(8):931-45.
8. Foa EB, Huppert JD, Leiberg S, Langner R, Kichic R, Hajcak G, . The Obsessive-Compulsive Inventory: development and validation of a short version. Psychological assessment. 2002;14(4):485.
9. Clayton AH, McGarvey EL, and Clavet GJ. The changes in sexual functioning questionnaire(CSFQ): Development, reliability and validity. Psychopharmacology Bulletin. 1997;33(4):731-745.
10. Caspi A, Sugden K, Moffitt TE, Taylor A, Craig IW, Harrington H, et al. Influence of life stress on depression: moderation by a polymorphism in the 5-HTT gene. Science. 2003;301(5631):386-9.
11. Meyer TJ, Miller ML, Metzger RL, Borkovec TD. Development and validation of the penn state worry questionnaire. Behaviour research and therapy. 1990;28(6):487-95.
12. Tangney JP, Boone AL, Baumeister RF. High self-control predicts good adjustment, less pathology, better grades, and interpersonal success. Journal of Personality. 2004;72(2):271-324.
13. Spielberger CD. State-trait anxiety inventory for adults. 1983.
14. Stewart-Brown S, Janmohamed K. Warwick-Edinburgh mental well-being scale. User guide. Version. 2008;1(10.1037).
15. Carlson LE, Brown KW. Validation of the Mindful Attention Awareness Scale in a cancer population. Journal of psychosomatic research. 2005;58(1):29-33.
16. Davis, M. H. A multidimensional approach to individual differences in empathy. JSAS Catalog of Selected Documents in Psychology. 1980;10;85.
17. McNair DM, Lorr, M, Droppleman LF. (1971) Manual for the Profile of Mood States. San Diego, CA: Educational and Industrial Testing Service.
18. Buysse DJ, Reynolds III CF, Monk TH, Berman SR, Kupfer DJ. The Pittsburgh Sleep Quality Index: a new instrument for psychiatric practice and research. Psychiatry research. 1989;28(2):193-213.
19. Derogatis LR. (1975). Brief Symptom Inventory. Baltimore, MD: Clinical Psychometric Research.
20. Norris H. The action of sedatives on brain stem oculomotor systems in man. Neuropharmacology. 1971;10(2):181-91.
21. Cohen S, Kamarck T, Mermelstein R. A global measure of perceived stress. Journal of health and social behavior. 1983;24:385-96.
22. Snyder‐Halpern R, Verran JA. Instrumentation to describe subjective sleep characteristics in healthy subjects. Research in nursing & health. 1987;10(3):155-63.
23. Murphy F, Smith K, Cowen P, Robbins T, Sahakian B. The effects of tryptophan depletion on cognitive and affective processing in healthy volunteers. Psychopharmacology. 2002;163(1):42-53.
24. Marzuki AA, Tomić I, Ip SH, Gottwald J, Kanen JW, Kaser M, et al. Association of environmental uncertainty with altered decision-making and learning mechanisms in youths with obsessive-compulsive disorder. JAMA network open. 2021;4(11):e2136195-.
25. Decker JH, Otto AR, Daw ND, Hartley CA. From creatures of habit to goal-directed learners: Tracking the developmental emergence of model-based reinforcement learning. Psychological science. 2016;27(6):848-58.
26. Skandali N, Rowe JB, Voon V, Deakin JB, Cardinal RN, Cormack F, et al. Dissociable effects of acute SSRI (escitalopram) on executive, learning and emotional functions in healthy humans. Neuropsychopharmacology. 2018;43(13):2645-51.
27. Ye Z, Altena E, Nombela C, Housden CR, Maxwell H, Rittman T, et al. Selective serotonin reuptake inhibition modulates response inhibition in Parkinson’s disease. Brain. 2014;137(4):1145-55.
28. Cambridge Cognition. https://www.cambridgecognition.com/
29. Rogers RD, Everitt BJ, Baldacchino A, Blackshaw AJ, Swainson R, Wynne K, et al. Dissociable deficits in the decision-making cognition of chronic amphetamine abusers, opiate abusers, patients with focal damage to prefrontal cortex, and tryptophan-depleted normal volunteers: evidence for monoaminergic mechanisms. Neuropsychopharmacology. 1999;20(4):322-39.
30. CamCops. <https://camcops.readthedocs.io/en/latest/tasks/ided3d.html#ided3d>
31. Sahakian BJ, Morris RG, Evenden JL, Heald A, Levy R, Philpot M, et al. A comparative study of visuospatial memory and learning in Alzheimer-type dementia and Parkinson's disease. Brain. 1988;111(3):695-718.
32. de Rover M, Pironti VA, McCabe JA, Acosta-Cabronero J, Arana FS, Morein-Zamir S, et al. Hippocampal dysfunction in patients with mild cognitive impairment: a functional neuroimaging study of a visuospatial paired associates learning task. Neuropsychologia. 2011;49(7):2060-70.
33. Owen AM, Downes JJ, Sahakian BJ, Polkey CE, Robbins TW. Planning and spatial working memory following frontal lobe lesions in man. Neuropsychologia. 1990;28(10):1021-34.
34. Coull JT, Middleton HC, Robbins TW, Sahakian BJ. Clonidine and diazepam have differential effects on tests of attention and learning. Psychopharmacology. 1995;120(3):322-32.
35. Bland AR, Roiser JP, Mehta MA, Schei T, Boland H, Campbell-Meiklejohn DK, et al. EMOTICOM: a neuropsychological test battery to evaluate emotion, motivation, impulsivity, and social cognition. Frontiers in Behavioral Neuroscience. 2016;10:25.
36. Kanen JW, Ersche KD, Fineberg NA, Robbins TW, Cardinal RN. Computational modelling reveals contrasting effects on reinforcement learning and cognitive flexibility in stimulant use disorder and obsessive-compulsive disorder: remediating effects of dopaminergic D2/3 receptor agents. Psychopharmacology. 2019;236(8):2337-58.
37. Den Ouden HE, Daw ND, Fernandez G, Elshout JA, Rijpkema M, Hoogman M, et al. Dissociable effects of dopamine and serotonin on reversal learning. Neuron. 2013;80(4):1090-100.
38. Gronau QF, Sarafoglou A, Matzke D, Ly A, Boehm U, Marsman M, et al. A tutorial on bridge sampling. Journal of mathematical psychology. 2017;81:80-97.
39. Gelman A, Hill J, Yajima M. Why we (usually) don't have to worry about multiple comparisons. Journal of research on educational effectiveness. 2012;5(2):189-211.
40. Kruschke JK. Bayesian data analysis. Wiley Interdisciplinary Reviews: Cognitive Science. 2010;1:658-76.

**Table S1**. *Neuropsychological Tests and Outcome Measures*

| Task | Outcome Measure | Pre-Registered Category | Domain | Task References |
| --- | --- | --- | --- | --- |
| Probabilistic Reversal Learning Task | Mean Errors Stage 1 | Primary | Learning | Chamberlain et al., 2006; Skandali et al. 2018 |
|  | Mean Errors Stage 2 | Primary | Learning |  |
|  | Stage 1 ProbSwitch | Secondary | Learning |  |
|  | Stage 2 ProbSwitch | Secondary | Learning |  |
| Probabilistic Reversal Learning Task | Hierarchical Bayesian Modelling Parameters | Primary | Reinforcement Behaviour | Kanen et al., 2019; Marzuki et al., 2021 |
| Sequential Model-Based/Model-Free | Proportion of Stay * Reward * Tranistion Type | Secondary | Learning | Decker et al., 2016 |
|  | Proportion of Stay * Reward | Secondary | Learning |  |
|  | Proportion of Stay * Reward * Transition Type * Group | Secondary | Learning |  |
|  | Proportion of Stay * Reward * Group | Secondary | Learning |  |
| Interleaved Stop Signal Go/No-Go Task | Stop Signal Reaction Time | Primary | Inhibition | Ye et al., 2014; Skandali et al. 2018 |
|  | Go RT | Secondary | Inhibition |  |
|  | Go Comission Errors | Secondary | Inhibition |  |
|  | No-Go Errors | Secondary | Inhibition |  |
| 3 Dimensional Intra-Extra Dimension Set Shifting Task | Extra Dimension Shift Errors | Primary | Executive Function | https://camcops.readthedocs.io/en/latest/tasks/ided3d.html#ided3d; Rogers et al., 1999 |
|  | Pre-ED Errors | Secondary | Learning |  |
|  | Total Errors Adj IED | Secondary | Executive Function |  |
| CANTAB PAL | FTMS PAL | Secondary | Memory | https://www.cambridgecognition.com/; Sahakian et al., 1988; De Rover et al., 2011 |
|  | Total Errors Adj PAL | Secondary | Memory |  |
| CANTAB SWM | Between Search Errors | Secondary | Memory | https://www.cambridgecognition.com/; Owen et al., 1990 |
|  | Strategy Score | Secondary | Executive Function |  |
| CANTAB RVP | A' | Secondary | Attention | https://www.cambridgecognition.com/; Coull et al., 1995 |
|  | Latency | Secondary | Attention |  |
|  | False Alarms | Secondary | Attention |  |
| EMOTICOM Emotion Recognition Eyes | Affective Bias for D' | Primary | Emotion Recognition | Bland et al., 2016 |
|  | D' for Emotion Recognition | Primary | Emotion Recognition |  |
|  | Affective Bias for Hit Rate | Secondary | Emotion Recognition |  |
|  | Hit Rate for Emotion Recognition | Secondary | Emotion Recognition |  |
| EMOTICOM Intensity Morphing | Affective Bias decreasing | Primary | Emotion Recognition | Bland et al., 2016 |
|  | Detection Threshold Decreasing Negative Emotions | Primary | Emotion Recognition |  |
|  | Affective Bias increasing | Secondary | Emotion Recognition |  |
|  | Detection Threshold Increasing Negative Emotions | Secondary | Emotion Recognition |  |
| EMOTICOM Moral Judgement Task | Agent Guilt Score | Primary | Social Cognition | Bland et al., 2016 |
|  | Agent Shame Score | Primary | Social Cognition |  |
| EMOTICOM Ultimatum Game | Proportion of Offers Accepted | Primary | Social Cognition | Bland et al., 2016 |
|  | Fairness Sensitivity UG | Primary | Social Cognition |  |
| EMOTICOM Cambridge Gambling Task | Quality of DM | Secondary | Decision-Making | Bland et al., 2016 |
|  | Risk Adjustment | Secondary | Decision-Making |  |
|  | Overall Bet | Secondary | Decision-Making |  |
|  | Deliberation Time | Secondary | Decision-Making |  |

***Table S2.*** *Group Comparison of Questionnaires at Baseline*

| Pre-Registered Category | Questionnaire Measure | Mean Placebo | Mean Escitalopram | SD Placebo | SD Escitalopram | df | t-value | p-value | Cohen’s d |
| --- | --- | --- | --- | --- | --- | --- | --- | --- | --- |
| Secondary | IRI Scale 3 - Empathic Concern | 20.09 | 19.25 | 4.21 | 4.68 | 64.00 | 0.77 | 0.45 | 0.19 |
| Secondary | STAI-Trait - Sum score | 33.97 | 33.59 | 8.97 | 6.06 | 64.00 | 0.20 | 0.84 | 0.05 |
| Secondary | Staxi-2(trait) Trait Anger scale | 15.88 | 15.34 | 3.23 | 2.88 | 64.00 | 0.71 | 0.48 | 0.18 |
| Secondary | BDI-II score: Total sum | 3.06 | 2.41 | 3.21 | 2.60 | 64.00 | 0.90 | 0.37 | 0.22 |
| Secondary | BIS-11 total score | 57.97 | 54.66 | 8.15 | 7.16 | 64.00 | 1.75 | 0.09 | 0.43 |
| Secondary | BSI-53 score: Global Severity Index | 0.18 | 0.15 | 0.18 | 0.12 | 64.00 | 0.70 | 0.49 | 0.18 |
| Secondary | OCI total sum score | 7.62 | 6.50 | 7.16 | 6.13 | 64.00 | 0.68 | 0.50 | 0.17 |
| Secondary | Total Mood Disturbance score | 1.18 | -0.69 | 11.91 | 10.40 | 64.00 | 0.68 | 0.50 | 0.17 |
| Secondary | PSQI global score | 3.85 | 3.91 | 1.69 | 1.65 | 64.00 | -0.13 | 0.90 | 0.04 |
| Secondary | STAI-State - Sum score | 24.85 | 24.72 | 4.43 | 4.14 | 64.00 | 0.13 | 0.90 | 0.03 |
| Secondary | Staxi-2(state) State Anger scale | 15.06 | 15.22 | 0.34 | 0.61 | 48.27 | -1.31 | 0.20 | 0.32 |
| Secondary | VAS-Norris total mean score | 48.75 | 48.95 | 3.56 | 3.39 | 64.00 | -0.24 | 0.82 | 0.06 |
| Other | IRI Scale 1 - Perspective Taking | 19.26 | 20.25 | 3.81 | 3.75 | 64.00 | -1.06 | 0.29 | 0.26 |
| Other | IRI Scale 2 - Fantasy | 17.29 | 18.34 | 5.55 | 4.68 | 64.00 | -0.83 | 0.41 | 0.20 |
| Other | IRI Scale 4 - Personal Distress | 11.06 | 11.19 | 3.80 | 3.74 | 64.00 | -0.14 | 0.89 | 0.03 |
| Other | Handedness (Edinburgh score) | 84.57 | 90.77 | 29.27 | 16.63 | 64.00 | -1.05 | 0.30 | 0.26 |
| Other | Education score individual | 4.24 | 4.13 | 1.37 | 1.15 | 64.00 | 0.31 | 0.76 | 0.09 |
| Other | Total education score | 16.18 | 16.13 | 1.47 | 1.50 | 64.00 | 0.31 | 0.89 | 0.03 |
| Other | Education score mom | 4.03 | 4.09 | 1.06 | 0.96 | 64.00 | 0.14 | 0.80 | 0.06 |
| Other | Education score dad | 3.94 | 3.91 | 1.30 | 1.15 | 64.00 | -0.26 | 0.91 | 0.02 |
| Other | Occasional smoker | 1.57 | 1.66 | 0.50 | 0.48 | 64.00 | 0.12 | 0.49 | 0.18 |
| Other | Exact alcohol units per week | 5.41 | 4.47 | 4.37 | 3.52 | 64.00 | -0.69 | 0.36 | 0.24 |
| Other | Used these drugs within last 12 months | 1.68 | 1.75 | 0.48 | 0.44 | 64.00 | -0.65 | 0.52 | 0.15 |
| Other | Activity level | 3.00 | 3.03 | 0.43 | 0.47 | 64.00 | -0.28 | 0.78 | 0.07 |
| Other | BIS/BAS score - Total Score | 19.79 | 20.41 | 3.25 | 3.57 | 64.00 | -0.73 | 0.47 | 0.18 |
| Other | BIS/BAS score – Drive | 11.35 | 10.94 | 1.74 | 1.92 | 64.00 | 0.92 | 0.36 | 0.22 |
| Other | BIS/BAS score - Fun Seeking | 11.62 | 11.22 | 1.74 | 2.39 | 56.43 | 0.78 | 0.44 | 0.19 |
| Other | BIS/BAS score - Reward Responsiveness | 17.18 | 16.53 | 1.62 | 1.26 | 64.00 | 1.51 | 0.14 | 0.45 |
| Other | CSFQ-14 Dimension 1: Pleasure | 3.82 | 3.56 | 0.80 | 1.27 | 51.61 | 0.99 | 0.33 | 0.25 |
| Other | CSFQ-14 Dimension 2: Desire-Frequency | 7.44 | 7.06 | 1.26 | 1.88 | 53.68 | 0.95 | 0.34 | 0.24 |
| Other | CSFQ-14 Dimension 3: Desire-Interest | 10.09 | 9.16 | 2.17 | 2.54 | 64.00 | 1.61 | 0.11 | 0.39 |
| Other | CSFQ-14 Dimension 4: Arousal/Erection | 11.79 | 11.44 | 2.12 | 2.65 | 64.00 | 0.61 | 0.55 | 0.15 |
| Other | CSFQ-14 Dimension 5: Orgasm/Ejaculation | 11.62 | 11.16 | 1.92 | 2.77 | 64.00 | 0.79 | 0.43 | 0.19 |
| Other | CSFQ-14 Phase 1: Desire | 17.53 | 16.22 | 2.97 | 4.00 | 64.00 | 1.52 | 0.13 | 0.37 |
| Other | CSFQ-14 Phase 2: Arousal | 11.79 | 11.44 | 2.12 | 2.65 | 64.00 | 0.61 | 0.55 | 0.15 |
| Other | CSFQ-14 Phase 3: Orgasm/Completion | 11.62 | 11.16 | 1.92 | 2.77 | 64.00 | 0.79 | 0.43 | 0.19 |
| Other | CSFQ-14 score: Total sum | 54.00 | 51.13 | 6.55 | 9.64 | 64.00 | 1.43 | 0.16 | 0.35 |
| Other | IUS Factor 1 - Prospective Anxiety | 28.35 | 27.84 | 7.74 | 5.83 | 64.00 | 0.30 | 0.77 | 0.07 |
| Other | IUS Factor 2 - Inhibitory Anxiety | 25.97 | 26.06 | 8.60 | 8.57 | 64.00 | -0.04 | 0.97 | 0.01 |
| Other | IUS score - Total sum | 54.32 | 53.91 | 15.25 | 13.37 | 64.00 | 0.12 | 0.91 | 0.03 |
| Other | MAAS score: Mean of all items | 4.22 | 4.15 | 0.78 | 0.57 | 64.00 | 0.41 | 0.68 | 0.10 |
| Other | Number of recent positive events | 11.00 | 12.00 | 4.73 | 4.61 | 64.00 | -0.87 | 0.39 | 0.21 |
| Other | Number of life time positive events | 5.71 | 5.59 | 2.28 | 1.48 | 57.00 | 0.24 | 0.81 | 0.06 |
| Other | Total number of positive life events | 16.71 | 17.59 | 5.50 | 4.79 | 64.00 | -0.70 | 0.49 | 0.17 |
| Other | PSWQ score - Total sum | 44.53 | 46.78 | 13.24 | 12.37 | 64.00 | -0.71 | 0.48 | 0.18 |
| Other | Self-control total score | 120.74 | 126.97 | 13.46 | 9.75 | 64.00 | -2.14 | 0.04 | 0.53 |
| Other | Staxi-2(trait) Trait Anger: Angry Temperament | 6.18 | 5.78 | 1.70 | 1.31 | 64.00 | 1.05 | 0.30 | 0.26 |
| Other | Staxi-2(trait) Trait Anger subscale: Angry Reaction | 7.12 | 7.06 | 1.95 | 1.68 | 64.00 | 0.12 | 0.90 | 0.03 |
| Other | Staxi-2(trait) Anger Expression-Out | 12.12 | 11.75 | 2.20 | 2.23 | 64.00 | 0.67 | 0.50 | 0.17 |
| Other | Staxi-2(trait) Anger Expression-In | 16.68 | 17.00 | 5.04 | 3.80 | 61.15 | -0.30 | 0.77 | 0.07 |
| Other | Staxi-2(trait) Anger Control-Out | 25.53 | 27.41 | 3.56 | 3.09 | 64.00 | -2.28 | 0.03 | 0.56 |
| Other | Staxi-2(trait) Anger Control-In | 22.56 | 23.38 | 4.38 | 3.85 | 64.00 | -0.80 | 0.43 | 0.20 |
| Other | Staxi-2(trait) Anger Expression Index | 28.71 | 25.97 | 8.24 | 7.85 | 64.00 | 1.38 | 0.17 | 0.34 |
| Other | Number of recent stressful events | 3.04 | 2.62 | 2.79 | 1.96 | 50.00 | 0.63 | 0.53 | 0.17 |
| Other | Number of life time stressful events | 1.15 | 0.77 | 1.38 | 0.77 | 50.00 | 1.25 | 0.22 | 0.34 |
| Other | Total number of stressful life events | 4.19 | 3.38 | 3.43 | 2.33 | 50.00 | 0.99 | 0.33 | 0.28 |
| Other | WEMWBS total score | 54.76 | 55.28 | 8.10 | 7.82 | 64.00 | -0.77 | 0.44 | 0.07 |
| Other | BIS-11 factor I: Attentional Impulsiveness | 13.21 | 12.50 | 2.78 | 1.93 | 64.00 | 1.19 | 0.24 | 0.30 |
| Other | BIS-11 factor II: Motor Impulsiveness | 20.82 | 20.13 | 3.65 | 3.20 | 64.00 | 0.83 | 0.41 | 0.20 |
| Other | BIS-11 factor III: Non-Planning Impulsiveness | 23.94 | 22.03 | 4.49 | 4.28 | 64.00 | 1.77 | 0.08 | 0.44 |
| Other | BSI-53 factor: Depression | 0.14 | 0.11 | 0.20 | 0.18 | 64.00 | 0.60 | 0.55 | 0.15 |
| Other | BSI-53 factor: Anxiety | 0.21 | 0.19 | 0.20 | 0.22 | 64.00 | 0.33 | 0.75 | 0.09 |
| Other | BSI-53 factor: Somatization | 0.13 | 0.07 | 0.22 | 0.10 | 45.50 | 1.42 | 0.16 | 0.34 |
| Other | BSI-53 factor: Psychoticism | 0.11 | 0.08 | 0.22 | 0.17 | 64.00 | 0.65 | 0.53 | 0.16 |
| Other | BSI-53 factor: Paranoid Ideation | 0.05 | 0.12 | 0.10 | 0.22 | 43.14 | -1.54 | 0.13 | 0.38 |
| Other | BSI-53 factor: Phobic Anxiety | 0.17 | 0.18 | 0.22 | 0.23 | 64.00 | -0.19 | 0.85 | 0.05 |
| Other | BSI-53 factor: Hostility | 0.11 | 0.07 | 0.14 | 0.12 | 64.00 | 1.15 | 0.26 | 0.28 |
| Other | BSI-53 factor: Obsession-Compulsion | 0.39 | 0.25 | 0.39 | 0.32 | 64.00 | 1.62 | 0.11 | 0.40 |
| Other | BSI-53 factor: Interpersonal Sensitivity | 0.20 | 0.23 | 0.31 | 0.27 | 64.00 | -0.39 | 0.70 | 0.10 |
| Other | BSI-53 score: Total number of positive symptoms | 7.50 | 6.78 | 6.34 | 5.11 | 64.00 | 0.51 | 0.62 | 0.13 |
| Other | BSI-53 score: Positive Symptoms Distress Index | 1.16 | 1.14 | 0.23 | 0.18 | 55.00 | 0.39 | 0.70 | 0.10 |
| Other | OCI subscale mean score - Washing | 0.12 | 0.12 | 0.20 | 0.24 | 64.00 | -0.06 | 0.95 | 0.01 |
| Other | OCI subscale mean score - Checking | 0.14 | 0.15 | 0.19 | 0.22 | 64.00 | -0.31 | 0.76 | 0.08 |
| Other | OCI subscale mean score - Doubting | 0.40 | 0.22 | 0.48 | 0.38 | 64.00 | 1.71 | 0.09 | 0.42 |
| Other | OCI subscale mean score – Ordering | 0.25 | 0.22 | 0.30 | 0.28 | 64.00 | 0.40 | 0.69 | 0.10 |
| Other | OCI subscale mean score - Obsessing | 0.13 | 0.09 | 0.18 | 0.16 | 64.00 | 0.75 | 0.45 | 0.18 |
| Other | OCI subscale mean score - Hoarding | 0.29 | 0.21 | 0.37 | 0.28 | 64.00 | 1.05 | 0.30 | 0.26 |
| Other | OCI subscale mean score - Mental Neutralising | 0.19 | 0.17 | 0.22 | 0.19 | 64.00 | 0.28 | 0.78 | 0.07 |
| Other | OCI total mean distress score | 0.18 | 0.16 | 0.17 | 0.15 | 64.00 | 0.68 | 0.50 | 0.16 |
| Other | Tension-Anxiety (T) factor | 5.35 | 5.28 | 2.40 | 2.28 | 64.00 | 0.12 | 0.90 | 0.03 |
| Other | Depression-Dejection (D) factor | 2.32 | 2.09 | 2.52 | 2.90 | 64.00 | 0.34 | 0.73 | 0.08 |
| Other | Anger-Hostility (A) factor | 1.85 | 2.19 | 2.45 | 2.66 | 64.00 | -0.53 | 0.60 | 0.13 |
| Other | Vigor-Activity (V) factor | 18.71 | 19.72 | 4.19 | 3.74 | 64.00 | -1.03 | 0.31 | 0.25 |
| Other | Fatigue-Inertia (F) factor | 5.18 | 4.66 | 3.41 | 3.76 | 64.00 | 0.59 | 0.56 | 0.14 |
| Other | Confusion-Bewilderment (C) factor | 5.18 | 4.81 | 2.32 | 1.94 | 64.00 | 0.69 | 0.49 | 0.17 |
| Other | PSQI: Subjective sleep quality | 0.76 | 0.78 | 0.50 | 0.55 | 64.00 | -0.13 | 0.90 | 0.04 |
| Other | PSQI: Sleep latency | 0.97 | 0.97 | 0.83 | 0.74 | 64.00 | 0.01 | 0.99 | 0.00 |
| Other | PSQI: Sleep duration | 0.09 | 0.06 | 0.38 | 0.25 | 64.00 | 0.33 | 0.75 | 0.09 |
| Other | PSQI: Habitual sleep efficiency | 0.24 | 0.53 | 0.43 | 0.76 | 48.36 | -1.93 | 0.06 | 0.47 |
| Other | PSQI: Sleep disturbances | 0.97 | 0.94 | 0.39 | 0.25 | 64.00 | 0.41 | 0.68 | 0.09 |
| Other | PSQI: Use of sleeping medication | 0.09 | 0.00 | 0.51 | 0.00 | 64.00 | 0.97 | 0.34 | 0.25 |
| Other | PSQI: Daytime dysfunction | 0.74 | 0.63 | 0.51 | 0.49 | 64.00 | 0.89 | 0.38 | 0.22 |
| Other | Staxi-2(state) State Anger: Feeling Angry | 5.06 | 5.16 | 0.34 | 0.45 | 64.00 | -1.00 | 0.32 | 0.25 |
| Other | Staxi-2(state) State Anger: Expr. Anger Verb. | 5.00 | 5.06 | 0.00 | 0.25 | 64.00 | -1.44 | 0.16 | 0.34 |
| Other | Staxi-2(state) State Anger: Expr. Anger Phys. | 5.00 | 5.00 | 0.00 | 0.00 | 64.00 | 0.00 | 1.00 | 0.00 |

***Table S3.*** *Group Comparison of Questionnaires Change Score between Baseline and Cognitive Visit.*

| Pre-Registered Category | Questionnaire Measure | Mean Placebo | Mean Escitalopram | SD Placebo | SD Escitalopram | df | t-value | p-value | Cohen's d |
| --- | --- | --- | --- | --- | --- | --- | --- | --- | --- |
| Secondary | IUS Factor 1 - Prospective Anxiety | -2.47 | -3.13 | 5.76 | 6.37 | 62.00 | 0.44 | 0.66 | 0.11 |
| Secondary | IUS Factor 2 - Inhibitory Anxiety | -1.29 | -2.30 | 4.77 | 5.52 | 62.00 | 0.78 | 0.44 | 0.20 |
| Secondary | IUS score - Total sum | -3.76 | -5.43 | 9.30 | 10.86 | 62.00 | 0.66 | 0.51 | 0.17 |
| Secondary | PSWQ score - Total sum | -1.88 | -3.90 | 7.33 | 7.42 | 62.00 | 1.09 | 0.28 | 0.27 |
| Secondary | Self-control total score | 3.56 | 3.60 | 7.79 | 11.15 | 62.00 | -0.02 | 0.99 | 0.00 |
| Secondary | CSFQ-14 Dimension 1: Pleasure | -0.38 | -0.47 | 1.13 | 1.28 | 62.00 | 0.28 | 0.78 | 0.07 |
| Secondary | CSFQ-14 Dimension 2: Desire-Frequency | -0.15 | -0.93 | 1.31 | 1.96 | 49.43 | 1.86 | 0.07 | 0.47 |
| Secondary | CSFQ-14 Dimension 3: Desire-Interest | -0.59 | -1.10 | 2.02 | 2.22 | 62.00 | 0.97 | 0.34 | 0.24 |
| Secondary | CSFQ-14 Dimension 4: Arousal/Erection | -0.35 | -0.63 | 1.32 | 1.99 | 62.00 | 0.67 | 0.51 | 0.17 |
| Secondary | CSFQ-14 Dimension 5: Orgasm/Ejaculation | 0.03 | -1.53 | 1.49 | 2.87 | 42.25 | 2.68 | 0.01* | 0.68 |
| Secondary | CSFQ-14 Phase 1: Desire | -0.74 | -2.03 | 3.01 | 3.82 | 54.97 | 1.50 | 0.14 | 0.38 |
| Secondary | CSFQ-14 Phase 2: Arousal | -0.47 | -0.63 | 1.46 | 1.99 | 62.00 | 0.38 | 0.71 | 0.09 |
| Secondary | CSFQ-14 Phase 3: Orgasm/Completion | 0.03 | -1.53 | 1.49 | 2.87 | 42.25 | 2.68 | 0.01* | 0.68 |
| Secondary | CSFQ-14 score: Total sum | -1.82 | -5.07 | 5.37 | 8.75 | 46.93 | 1.76 | 0.09 | 0.45 |
| Secondary | MAAS score: Mean of all items | 0.04 | 0.10 | 0.43 | 0.51 | 62.00 | -0.49 | 0.62 | 0.13 |
| Secondary | WEMWBS total score | -1.44 | -0.77 | 5.96 | 7.49 | 62.00 | -0.40 | 0.69 | 0.10 |
| Secondary | BDI-II score: Total sum | 1.32 | 1.20 | 4.56 | 3.36 | 62.00 | 0.12 | 0.90 | 0.03 |
| Secondary | BIS-11 factor I: Attentional Impulsiveness | -0.21 | 0.57 | 1.92 | 2.50 | 54.13 | -1.37 | 0.18 | 0.35 |
| Secondary | BIS-11 factor II: Motor Impulsiveness | 0.09 | 0.03 | 2.38 | 2.40 | 62.00 | 0.09 | 0.93 | 0.03 |
| Secondary | BIS-11 factor III: Non-Planning Impulsiveness | 0.85 | 0.37 | 2.74 | 3.24 | 62.00 | 0.65 | 0.52 | 0.16 |
| Secondary | BIS-11 total score | 0.74 | 0.97 | 3.28 | 5.64 | 45.35 | -0.20 | 0.84 | 0.05 |
| Secondary | BSI-53 factor: Depression | 0.12 | 0.04 | 0.38 | 0.19 | 50.81 | 1.14 | 0.26 | 0.28 |
| Secondary | BSI-53 factor: Anxiety | 0.01 | 0.01 | 0.24 | 0.20 | 62.00 | -0.12 | 0.99 | 0.00 |
| Secondary | BSI-53 factor: Somatization | 0.02 | 0.12 | 0.26 | 0.22 | 62.00 | -1.62 | 0.11 | 0.41 |
| Secondary | BSI-53 factor: Psychoticism | 0.01 | 0.06 | 0.23 | 0.20 | 62.00 | -0.99 | 0.32 | 0.25 |
| Secondary | BSI-53 factor: Paranoid Ideation | 0.06 | -0.01 | 0.17 | 0.18 | 62.00 | 1.49 | 0.14 | 0.37 |
| Secondary | BSI-53 factor: Phobic Anxiety | 0.04 | -0.07 | 0.30 | 0.13 | 46.35 | 1.88 | 0.07 | 0.46 |
| Secondary | BSI-53 factor: Hostility | 0.07 | 0.03 | 0.22 | 0.19 | 62.00 | 0.86 | 0.40 | 0.22 |
| Secondary | BSI-53 factor: Obsession-Compulsion | 0.08 | 0.13 | 0.56 | 0.44 | 62.00 | -0.40 | 0.69 | 0.10 |
| Secondary | BSI-53 factor: Interpersonal Sensitivity | 0.07 | -0.07 | 0.37 | 0.28 | 62.00 | 1.62 | 0.11 | 0.41 |
| Secondary | BSI-53 score: Global Severity Index | 0.05 | 0.03 | 0.22 | 0.14 | 62.00 | 0.43 | 0.67 | 0.11 |
| Secondary | BSI-53 score: Total number of positive symptoms | 1.24 | 0.09 | 5.84 | 4.39 | 62.00 | 0.26 | 0.80 | 0.22 |
| Secondary | BSI-53 score: Positive Symptoms Distress Index | 0.05 | 0.00 | 0.29 | 0.28 | 53.00 | 0.68 | 0.50 | 0.18 |
| Secondary | OCI subscale mean score - Washing | 0.02 | 0.02 | 0.21 | 0.22 | 62.00 | -0.04 | 0.97 | 0.01 |
| Secondary | OCI subscale mean score - Checking | 0.03 | 0.03 | 0.23 | 0.27 | 62.00 | 0.06 | 0.96 | 0.01 |
| Secondary | OCI subscale mean score - Doubting | -0.13 | -0.07 | 0.47 | 0.27 | 53.48 | -0.64 | 0.52 | 0.16 |
| Secondary | OCI subscale mean score – Ordering | -0.07 | -0.05 | 0.27 | 0.19 | 62.00 | -0.31 | 0.76 | 0.08 |
| Secondary | OCI subscale mean score - Obsessing | -0.01 | -0.02 | 0.19 | 0.18 | 62.00 | 0.12 | 0.90 | 0.03 |
| Secondary | OCI subscale mean score - Hoarding | -0.12 | -0.06 | 0.28 | 0.26 | 62.00 | -0.90 | 0.37 | 0.23 |
| Secondary | OCI subscale mean score - Mental Neutralising | -0.06 | -0.06 | 0.17 | 0.16 | 62.00 | -0.06 | 0.95 | 0.02 |
| Secondary | OCI total mean distress score | -0.03 | -0.02 | 0.15 | 0.12 | 62.00 | -0.29 | 0.78 | 0.07 |
| Secondary | OCI total sum score | -1.15 | -0.73 | 6.27 | 5.17 | 62.00 | -0.29 | 0.78 | 0.07 |
| Secondary | Tension-Anxiety (T) factor | 0.06 | 0.07 | 2.44 | 2.00 | 62.00 | 0.98 | 0.33 | 0.00 |
| Secondary | Depression-Dejection (D) factor | 1.76 | 0.50 | 5.84 | 3.97 | 62.00 | 1.00 | 0.32 | 0.25 |
| Secondary | Anger-Hostility (A) factor | 1.26 | 0.30 | 3.97 | 3.25 | 62.00 | 1.06 | 0.30 | 0.26 |
| Secondary | Vigor-Activity (V) factor | -0.38 | 0.07 | 5.60 | 5.03 | 62.00 | -0.34 | 0.74 | 0.08 |
| Secondary | Fatigue-Inertia (F) factor | 1.47 | 2.23 | 4.76 | 4.78 | 62.00 | -0.64 | 0.53 | 0.16 |
| Secondary | Confusion-Bewilderment (C) factor | 0.74 | 1.27 | 3.23 | 2.77 | 62.00 | -0.70 | 0.49 | 0.18 |
| Secondary | Total Mood Disturbance (TMD) score | 6.24 | 4.30 | 21.16 | 12.04 | 62.00 | 0.43 | 0.67 | 0.11 |
| Secondary | PSQI: Subjective sleep quality | -0.09 | 0.03 | 0.38 | 0.56 | 62.00 | -1.03 | 0.31 | 0.25 |
| Secondary | PSQI: Sleep latency | 0.03 | -0.13 | 0.58 | 0.63 | 62.00 | 1.08 | 0.28 | 0.27 |
| Secondary | PSQI: Sleep duration | -0.03 | 0.00 | 0.30 | 0.26 | 62.00 | -0.42 | 0.68 | 0.11 |
| Secondary | PSQI: Habitual sleep efficiency | 0.15 | -0.20 | 0.66 | 0.55 | 62.00 | 2.27 | 0.03 | 0.58 |
| Secondary | PSQI: Sleep disturbances | 0.03 | 0.03 | 0.46 | 0.41 | 62.00 | -0.04 | 0.97 | 0.00 |
| Secondary | PSQI: Use of sleeping medication | -0.09 | 0.00 | 0.51 | 0.00 | 62.00 | -0.94 | 0.35 | 0.25 |
| Secondary | PSQI: Daytime dysfunction | 0.06 | 0.10 | 0.55 | 0.89 | 47.16 | -0.22 | 0.82 | 0.05 |
| Secondary | PSQI global score | 0.06 | -0.17 | 1.56 | 1.76 | 62.00 | 0.54 | 0.59 | 0.14 |
| Secondary | STAI-State - Sum score | 2.32 | 0.73 | 5.11 | 5.15 | 62.00 | 1.24 | 0.22 | 0.31 |
| Secondary | Staxi-2(state) State Anger scale | 0.26 | -0.10 | 1.26 | 0.48 | 62.00 | 1.49 | 0.14 | 0.38 |
| Secondary | Staxi-2(state) State Anger: Feeling Angry | 0.21 | -0.07 | 0.95 | 0.37 | 62.00 | 1.48 | 0.14 | 0.39 |
| Secondary | Staxi-2(state) State Anger: Expr. Anger Verb. | 0.06 | -0.03 | 0.34 | 0.18 | 62.00 | 1.32 | 0.19 | 0.33 |
| Secondary | Staxi-2(state) State Anger: Expr. Anger Phys. | 0.00 | 0.00 | 0.00 | 0.00 | 62.00 | 0.00 | 1.00 | 0.00 |
| Secondary | VAS-Norris total mean score | 0.36 | 0.65 | 3.32 | 3.58 | 62.00 | -0.34 | 0.74 | 0.08 |

*Note: * survives FDR correction.*

***Table S4.*** *Group Comparison of Questionnaires at Cognitive Visit.*

| Pre-Registered Category | Questionnaire Measure | Mean Placebo | Mean Escitalopram | SD Placebo | SD Escitalopram | df | t-value | p-value | Cohen's d |
| --- | --- | --- | --- | --- | --- | --- | --- | --- | --- |
| Secondary | Cohen PSS | 1.41 | 0.33 | 3.77 | 3.93 | 62.00 | 1.12 | 0.27 | 0.28 |
| Secondary | VSH domain score - Disturbance | 80.13 | 79.12 | 16.51 | 17.18 | 62.00 | 0.24 | 0.81 | 0.06 |
| Secondary | VSH domain score - Effectiveness | 82.17 | 76.38 | 13.94 | 14.68 | 62.00 | 1.62 | 0.11 | 0.40 |
| Secondary | VSH - Supplementary | 78.99 | 67.76 | 21.47 | 26.89 | 62.00 | 1.86 | 0.07 | 0.46 |
| Secondary | VSH total score | 79.58 | 75.45 | 12.96 | 12.48 | 62.00 | 1.29 | 0.20 | 0.32 |

***Table S5.*** *Group Comparison of Questionnaires at 1 week after cognitive visit.*

| Pre-Registered Category | Questionnaire Measure | Mean Placebo | Mean Drug | SD Placebo | SD Escitalopram | df | t-value | p-value | Cohen's d |
| --- | --- | --- | --- | --- | --- | --- | --- | --- | --- |
| Other | BIS-11 factor I: Attentional Impulsiveness | 13.00 | 12.73 | 3.15 | 2.56 | 59.00 | 0.36 | 0.72 | 0.09 |
| Other | BIS-11 factor II: Motor Impulsiveness | 20.61 | 20.20 | 3.68 | 2.86 | 59.00 | 0.49 | 0.63 | 0.12 |
| Other | BIS-11 factor III: Non-Planning Impulsiveness | 23.74 | 23.03 | 5.09 | 5.18 | 59.00 | 0.54 | 0.59 | 0.14 |
| Other | BIS-11 total score | 57.35 | 55.97 | 9.56 | 7.90 | 59.00 | 0.62 | 0.54 | 0.16 |
| Other | VAS-Norris total mean score | 49.63 | 49.81 | 3.45 | 2.36 | 53.10 | -0.23 | 0.82 | 0.06 |
| Other | Tension-Anxiety (T) factor | 5.74 | 5.13 | 2.56 | 3.52 | 59.00 | 0.78 | 0.44 | 0.20 |
| Other | Depression-Dejection (D) factor | 3.45 | 3.43 | 4.03 | 5.85 | 59.00 | 0.01 | 0.99 | 0.00 |
| Other | Anger-Hostility (A) factor | 2.68 | 3.53 | 2.56 | 4.58 | 45.18 | -0.90 | 0.38 | 0.23 |
| Other | Vigor-Activity (V) factor | 18.87 | 19.90 | 5.16 | 6.24 | 59.00 | -0.70 | 0.49 | 0.18 |
| Other | Fatigue-Inertia (F) factor | 6.03 | 4.90 | 4.89 | 4.57 | 59.00 | 0.93 | 0.35 | 0.24 |
| Other | Confusion-Bewilderment (C) factor | 5.42 | 5.27 | 2.54 | 2.97 | 59.00 | 0.22 | 0.83 | 0.05 |
| Other | Total Mood Disturbance (TMD) score | 4.45 | 2.37 | 16.50 | 22.20 | 59.00 | 0.42 | 0.68 | 0.11 |
| Other | Staxi-2(state) State Anger scale | 15.68 | 16.67 | 1.14 | 4.92 | 31.99 | -1.07 | 0.29 | 0.28 |
| Other | Staxi-2(state) State Anger: Feeling Angry | 5.58 | 5.97 | 0.85 | 2.41 | 35.84 | -0.84 | 0.42 | 0.22 |
| Other | Staxi-2(state) State Anger: Expr. Anger Verb. | 5.10 | 5.47 | 0.54 | 1.61 | 35.20 | -1.19 | 0.24 | 0.31 |
| Other | Staxi-2(state) State Anger: Expr. Anger Phys. | 5.00 | 5.23 | 0.00 | 0.97 | 29.00 | -1.32 | 0.20 | 0.33 |

***Table S6.*** *Model Comparison for the PRL Modelling.*

| Model | Model Rank | Parameters | Log Marginal Likelihood | Maximum R-hat |
| --- | --- | --- | --- | --- |
| 1 | 2 | α_rew_, α_pun_, τ_reinf_ | -1107.97 | 1.001794622 |
| 2 | 1 | α_rew_, α_pun_, τ_reinf_, τ_stim_ | -1107.64 | 1.006613078 |
| 3 | 4 | α, τ_reinf_, τ_stim_ | -1167.6 | 1.263413171 |
| 4 | 3 | ρ, φ, β | -1122.87 | 1.002992 |

***Table S7.*** *Group Comparison for standard PRL and MBMF measures.*

| Pre-Registered Category | Domain | Task | Outcome Measure | df | Estimate | Std Error | t -Value | p-Value | 95% Confidence Interval | |
| --- | --- | --- | --- | --- | --- | --- | --- | --- | --- | --- |
| Primary | Learning | Probabilistic Reversal Learning Task | Mean Errors Stage 1 | 5,60 | 0.010 | 0.007 | 1.444 | 0.154 | -0.004 | 0.024 |
|  |  |  | Mean Errors Stage 2 | 5,60 | -0.005 | 0.015 | -0.337 | 0.737 | -0.034 | 0.024 |
| Secondary | Learning | Probabilistic Reversal Learning Task | Stage 1 ProbSwitch | 5,60 | 0.006 | 0.003 | 1.967 | 0.054 | 0.000 | 0.012 |
|  |  |  | Stage 2 ProbSwitch | 5,60 | 0.002 | 0.002 | 0.614 | 0.541 | -0.002 | 0.006 |
|  |  | Sequential Model-Based/Model-Free Task | Proportion of Stay ~ Reward * Transition Type | 5,60 | 0.878 | 0.279 | 3.149 | 0.002 | 0.331 | 1.425 |
|  |  |  | Proportion of Stay ~ Reward | 5,60 | 1.019 | 0.189 | 5.382 | 0.00 | 0.648 | 1.390 |
|  |  |  | Proportion of Stay ~ Reward * Transition Type * Group | 5,60 | -0.167 | 0.157 | -1.063 | 0.288 | -0.475 | 0.141 |
|  |  |  | Proportion of Stay ~ Reward * Group | 5,60 | -0.336 | 0.117 | -2.873 | 0.004 | -0.565 | -0.107 |

***Table S8.*** *Group Comparison for ‘hot’ cognitive measures.*

| Domain | Task | Outcome Measure | Mean Placebo | Mean Escitalopram | SD Placebo | SD Escitalopram | df | Estimate | Std Error | t-Value | p-Value | 95% Confidence Interval | |
| --- | --- | --- | --- | --- | --- | --- | --- | --- | --- | --- | --- | --- | --- |
| Emotion Recognition  (Primary) | EMOTICOM Intensity Morphing | Affective Bias decreasing | -0.03 | -0.04 | 0.18 | 0.18 | 5,60 | -0.01 | 0.05 | -0.12 | 0.91 | -0.10 | 0.09 |
|  |  | Detection Threshold Decreasing Negative Emotions | 0.49 | 0.49 | 0.14 | 0.15 | 5,60 | 0.01 | 0.04 | 0.15 | 0.88 | -0.06 | 0.07 |
|  | EMOTICOM Emotion Recognition | Affective Bias for D' | -0.09 | 0.10 | 1.31 | 1.11 | 5,60 | 0.19 | 0.30 | 0.64 | 0.52 | -0.40 | 0.78 |
|  |  | D' for Emotion Recognition | -1.01 | 0.11 | 1.84 | 2.18 | 5,60 | 0.14 | 0.51 | 0.27 | 0.79 | -0.87 | 1.15 |
| Social Cognition  (Primary) | EMOTICOM Moral Judgement | Agent Guilt Score | 6.34 | 6.15 | 0.62 | 0.71 | 5,60 | -0.19 | 0.17 | -1.11 | 0.27 | -0.52 | 0.14 |
|  |  | Agent Shame Score | 6.03 | 5.88 | 0.98 | 0.75 | 5,60 | -0.15 | 0.22 | -0.66 | 0.51 | -0.58 | 0.29 |
|  | EMOTICOM Ultimatum Game | Proportion of Offers Accepted | 54.76 | 54.99 | 23.20 | 17.00 | 5,60 | -0.05 | 0.05 | -0.94 | 0.35 | -0.14 | 0.05 |
| Emotion Recognition  (Secondary) | EMOTICOM Intensity Morphing | Affective Bias increasing | 0.11 | 0.03 | 0.13 | 0.15 | 5,60 | -0.08 | 0.04 | -2.17 | 0.03 | -0.15 | -0.01 |
|  |  | Detection Threshold Increasing Negative Emotions | 0.55 | 0.55 | 0.10 | 0.11 | 5,60 | 0.00 | 0.03 | 0.00 | 1.00 | -0.05 | 0.05 |
|  | EMOTICOM Emotion Recognition | Affective Bias for Hit Rate | 0.00 | -0.03 | 0.18 | 0.22 | 5,60 | -0.01 | 0.05 | -0.28 | 0.78 | -0.11 | 0.08 |
|  |  | Hit Rate for Emotion Recognition | 0.74 | 0.74 | 0.07 | 0.08 | 5,60 | 0.01 | 0.02 | 0.27 | 0.79 | -0.03 | 0.04 |
| Social Cognition  (Secondary) | EMOTICOM Ultimatum Game | Fairness Sensitivity UG | 5.08 | 4.87 | 3.16 | 1.77 | 5,60 | 0.01 | 0.01 | 1.01 | 0.32 | 0.00 | 0.01 |
| Decision-Making  (Secondary) | EMOTICOM Cambridge Gambling Task | Quality of DM | 97.74 | 97.33 | 3.59 | 4.83 | 5,55 | -0.01 | 0.01 | -0.53 | 0.60 | -0.03 | 0.02 |
|  |  | Risk Adjustment | 0.59 | 0.56 | 0.46 | 0.42 | 5,55 | -0.01 | 0.00 | -0.79 | 0.43 | -0.01 | -0.01 |
|  |  | Overall Bet | 2.47 | 2.49 | 0.11 | 0.09 | 5,55 | 0.00 | 0.00 | -0.02 | 0.99 | 0.00 | 0.00 |
|  |  | Deliberation Time | 2172.00 | 2122.00 | 781.85 | 683.47 | 5,55 | 0.00 | 0.00 | 0.75 | 0.46 | 0.00 | 0.00 |

***Table S9.*** *Group Comparison for ‘cold’ cognitive measures.*

| Domain | Task | Outcome Measure | Mean Placebo | Mean Escitalopram | SD Placebo | SD Escitalopram | df | Estimate | Std Error | t-Value | p-Value | 95% Confidence Interval | |
| --- | --- | --- | --- | --- | --- | --- | --- | --- | --- | --- | --- | --- | --- |
| Inhibition (Primary) | Interleved Stop Signal Go/No-Go | SSRT | 342.32 | 348.73 | 26.42 | 32.17 | 5,48 | 0.15 | 0.08 | 1.83 | 0.07 | -0.01 | 0.30 |
| Executive Function  (Primary) | 3 Dimensional Intra-Extra Dimension Set Shifting Task | ED Shift Errors | 8.52 | 5.28 | 9.20 | 6.28 | 5,59 | 0.02 | 0.02 | 0.87 | 0.39 | -0.02 | 0.05 |
| Learning  (Secondary) | 3 Dimensional Intra-Extra Dimension Set Shifting Task | Pre-ED Errors | 8.42 | 7.41 | 7.23 | 6.04 | 5,59 | 0.00 | 0.02 | 0.12 | 0.90 | -0.03 | 0.03 |
| Inhibition  (Secondary) | Interleved Stop Signal Go/No-Go | Go RT | 474.17 | 463.08 | 77.97 | 56.97 | 5,48 | 0.25 | 0.17 | 1.44 | 0.16 | -0.09 | 0.58 |
|  |  | Go Comission Errors | 13.25 | 9.96 | 20.11 | 6.58 | 5,48 | 0.01 | 0.03 | 0.26 | 0.80 | -0.04 | 0.06 |
|  |  | No-Go Errors | 5.89 | 4.65 | 5.55 | 3.48 | 5,48 | -0.02 | 0.01 | -1.40 | 0.17 | -0.04 | 0.01 |
| Executive Function  (Secondary) | 3 Dimensional Intra-Extra Dimension Set Shifting Task | Total Errors Adj IED | 26.53 | 20.97 | 27.36 | 24.60 | 5,60 | 0.05 | 0.06 | 0.78 | 0.44 | -0.07 | 0.17 |
|  | CANTAB SWM | Strategy Score SWM | 29.38 | 32.69 | 16.41 | 14.70 | 5,60 | 0.03 | 0.04 | 0.81 | 0.42 | -0.04 | 0.10 |
| Memory  (Secondary) | CANTAB PAL | FTMS PAL | 28.00 | 30.71 | 7.36 | 5.17 | 5,59 | 0.01 | 0.02 | 0.82 | 0.42 | -0.02 | 0.04 |
|  |  | Total Errors Adj PAL | 18.76 | 10.81 | 18.17 | 8.06 | 5,59 | -0.01 | 0.03 | -0.32 | 0.75 | -0.06 | 0.05 |
|  | CANTAB SWM | Between Search Errors SWM | 50.82 | 52.94 | 46.37 | 44.07 | 5,60 | 0.05 | 0.10 | 0.50 | 0.62 | -0.15 | 0.25 |
|  |  |  |  |  |  |  |  |  |  |  |  |  |  |
| Attention  (Secondary) | CANTAB RVP | A' RVP | 0.93 | 0.92 | 0.04 | 0.04 | 5,60 | 0.00 | 0.00 | -1.32 | 0.19 | 0.00 | 0.00 |
|  |  | Latency RVP | 411.21 | 427.23 | 43.98 | 70.24 | 5,60 | 0.01 | 0.13 | 0.07 | 0.94 | -0.25 | 0.27 |
|  |  | False Alarms RVP | 2.41 | 3.34 | 1.74 | 2.52 | 5,60 | 0.00 | 0.01 | 0.42 | 0.67 | -0.01 | 0.01 |
