# Supplementary figures and images for: Chronic escitalopram in healthy volunteers has specific effects on reinforcement sensitivity: a double-blind, placebo-controlled semi-randomised study
Source: Neuropsychopharmacology. 2023 Jan 23;48(4):664–70. doi: 10.1038/s41386-022-01523-x (PMC9938113; doi:10.1038/s41386-022-01523-x)

## CONSORT 2010 Flow Diagram

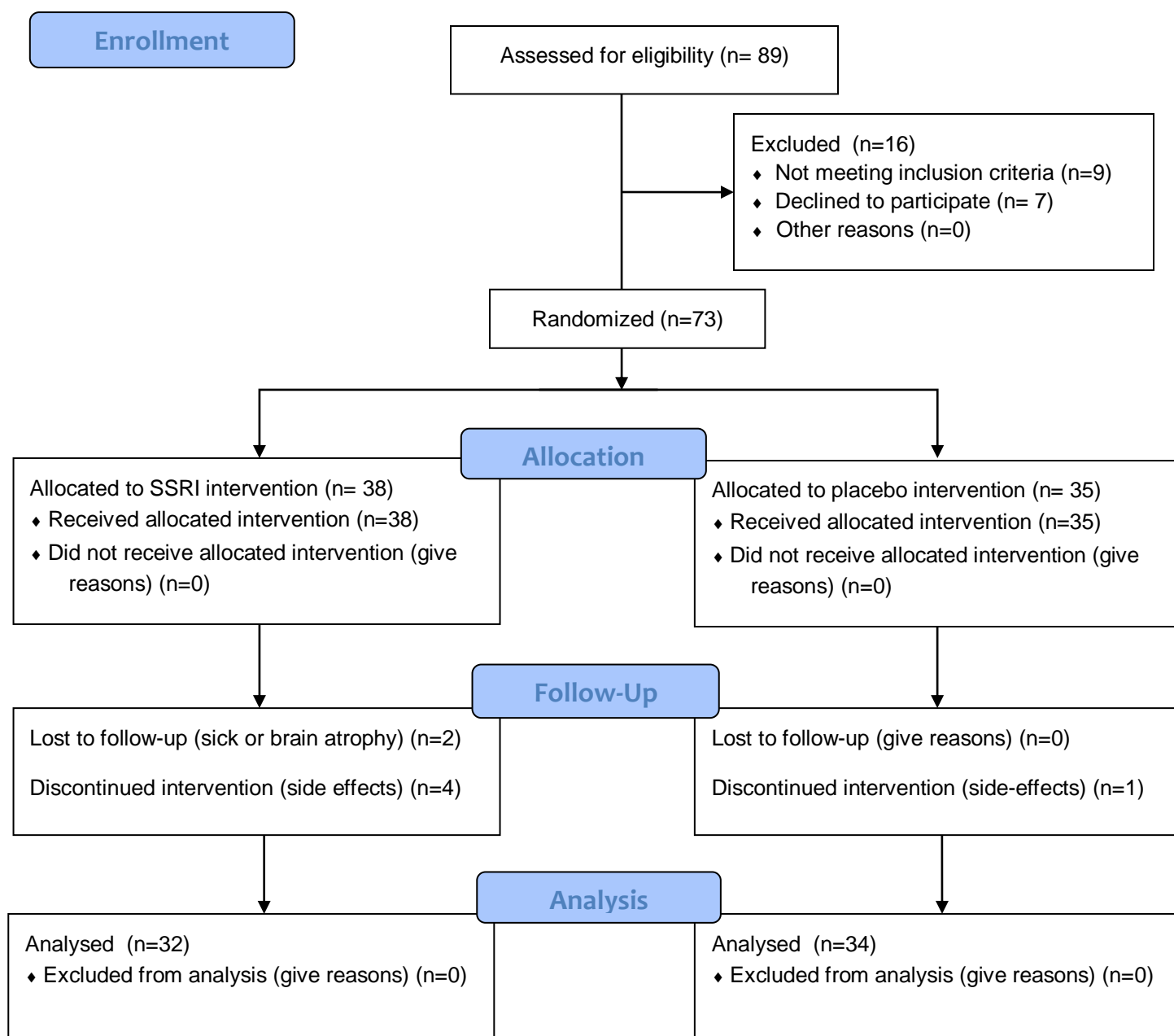

Supplement: Supplementary file 2 — CONSORT Flow Diagram [file 41386_2022_1523_MOESM2_ESM.pdf]
